# Eave tubes for malaria control in Africa: a modelling assessment of potential impact on transmission

**DOI:** 10.1186/s12936-016-1505-1

**Published:** 2016-09-02

**Authors:** Jessica L. Waite, Penelope A. Lynch, Matthew B. Thomas

**Affiliations:** 1 Department of Entomology, Center for Infectious Disease Dynamics, Pennsylvania State University, Merkle Building, University Park, PA 16802 USA; 2 College of Life & Environmental Sciences, University of Exeter, Penryn Campus, Cornwall, TR10 9FE UK

**Keywords:** Eave tube, Novel intervention, Vector control, Malaria, Housing, Eaves, *Anopheles*, Population model

## Abstract

**Background:**

Novel interventions for malaria control are necessary in the face of problems such as increasing insecticide resistance and residual malaria transmission. One way to assess performance prior to deployment in the field is through mathematical modelling. Modelled here are a range of potential outcomes for eave tubes, a novel mosquito control tool combining house screening and targeted use of insecticides to provide both physical protection and turn the house into a lethal mosquito killing device.

**Methods:**

The effect of eave tubes was modelled by estimating the reduction of infectious mosquito bites relative to no intervention (a transmission metric defined as relative transmission potential, RTP). The model was used to assess how RTP varied with coverage when eave tubes were used as a stand-alone intervention, or in combination with either bed nets (LLINs) or indoor residual spraying (IRS).

**Results:**

The model indicated the impact of eave tubes on transmission increases non-linearly as coverage increases, suggesting a community level benefit. For example, based on realistic assumptions, just 30 % coverage resulted in around 70 % reduction in overall RTP (i.e. there was a benefit for those houses without eave tubes). Increasing coverage to around 70 % reduced overall RTP by >90 %. Eave tubes exhibited some redundancy with existing interventions, such that combining interventions within properties did not give reductions in RTP equal to the sum of those provided by deploying each intervention singly. However, combining eave tubes and either LLINs or IRS could be extremely effective if the technologies were deployed in a non-overlapping way.

**Conclusion:**

Using predictive models to assess the benefit of new technologies has great value, and is especially pertinent prior to conducting expensive, large scale, randomized controlled trials. The current modelling study indicates eave tubes have considerable potential to impact malaria transmission if deployed at scale and can be used effectively with existing tools, especially if they are combined strategically with, for example, IRS and eave tubes targeting different houses.

**Electronic supplementary material:**

The online version of this article (doi:10.1186/s12936-016-1505-1) contains supplementary material, which is available to authorized users.

## Background

Wide scale use of mosquito control interventions, such as indoor residual spraying (IRS) and long-lasting insecticide-treated bed nets (LLINs), have made a major contribution to the substantial decline in malaria burden observed over the last decade [1]. However, new mosquito control tools are now required to address problems of insecticide resistance and residual transmission (i.e. the malaria transmission persisting following universal coverage of existing effective interventions such as IRS and/or LLINs) [2, 3].

Numerous studies show that house screening can reduce entry of mosquitoes [4–8] and can impact transmission [9]. Other studies find better housing correlates with reductions in malaria, particularly if eaves are closed or screened to prevent *Anopheles* mosquitoes from entering [10–14].

Eave tubes (see [15] for an introduction to the technology) represent a novel twist on the house modification approach. When referring to “eave tubes”, this is actually shorthand for a package of house modification wherein windows are screened, open eaves are closed and tubes (pieces of PVC piping) are installed into the eaves at 1–2 m intervals. These open eave tubes are fitted with electrostatic netting [16] treated with an insecticidal active, and so when mosquitoes are drawn toward the odours emanating from the house and attempt to enter through the eaves, they are killed. The electrostatic coating on the netting provides the additional advantage of increasing the bioavailability of powdered insecticides, delivering a lethal dose of insecticide even following transient contact [16, 17]. The netting can be used with diverse classes of insecticidal powders ranging from chemicals currently approved for IRS through to novel actives such as entomopathogenic fungi [17]. Fitting a house with eave tubes in effect turns the house into a mosquito-killing device.

To date, studies with eave tubes have centered around laboratory and semi-field investigations providing insights into potential effects at small scale [15, 18, Snetselaar et al. *pers. comm.*]. Where the technology has been deployed at larger scale the focus of studies has been on operational questions of feasibility of implementation and user acceptance [15]. Thus far, there is little understanding of how eave tubes are likely to affect entomological or epidemiological outcomes when deployed at scale and/or in combination with existing control tools such as IRS or LLINs. The aim of the current study is to use a population model to help bridge this knowledge gap.

## Methods

**Fig. 1:**
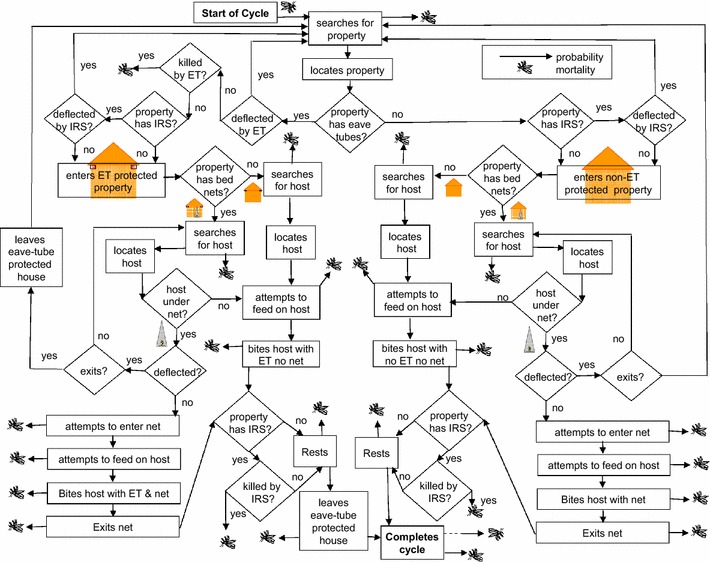
Model structure is summarized in this flow diagram, beginning with a mosquito entering the model and searching for a property, and ending with the completion of a cycle

A simple deterministic model was developed to assess the effects of IRS, LLINs and eave tubes on the average number of infectious bites per vector per lifetime, Because many relevant vector life-history parameters are still not well quantified in the field, the results are presented in terms of comparison between values calculated by the model assuming specific interventions and those calculated assuming no intervention, minimizing the impact of non-intervention related parameter values on the conclusions. A wide range of values for key parameters was used to generate results. The model is based on probabilities of given events during the mosquito gonotrophic cycle, and considers mortality or deflection to other properties before entering an eave tube protected property, and mortality or deflection, with a probability of exiting the property, when encountering LLINs within a property, as well as mortality while resting in IRS treated properties. In this instance deflection means that a host-seeking vector is deterred from attempting to enter a selected property, or attempting to reach a selected host under a bed net, and instead returns to searching behaviours. The model structure is summarized in Fig. 1 and the model is given in full in supplementary materials (Additional file 1, Eave tube simple model). The results were generated using a version of the model executed using excel.

The model makes a number of simplifying assumptions. Mosquitoes that commence host-seeking are assumed to feed or die during one night. Non-human feeding and multiple feeds during one gonotrophic cycle are ignored. Vector mortality is assumed to be unaffected by vector age or infection status. Average bite rate and length of parasite extrinsic incubation period are assumed to be constant between vectors and over time. Mosquitoes locate properties and hosts within properties randomly. There is no difference in the average number of people per property in properties with and without interventions, so an intervention applied to a given proportion of properties is also applied to that proportion of the human population.

The baseline parameter values used in the analysis are summarized in Table 1. Sensitivity analyses were conducted for the non-intervention related parameters. Although variation around the selected values in some instances produced quantitative changes in the results, they did not affect the conclusions (see Additional file 2, Sensitivity analysis), and the key metric is intentionally formulated in comparative terms to minimize the impact of chosen values for parameters common to all intervention types.

**Table 1:** Table of baseline parameter values used by model unless otherwise indicated

Description	Value	Units
Assumed cycle length	3.00^a^	Days
Average search time to locate a property	0.50^b^	Hours
Search time to locate a human host when searching indoors	0.25^b^	Hours
Average time spent resting indoors post-feed	8.00^b^	Hours
Average time spent finding ovipositing site	8.00^b^	Hours
Average time spent from ovipositing to host searching	55.25^b^	Hours
Base mortality rate while searching for property or laying site	10.00 %^a^	Instantaneous daily rate
Base mortality rate while searching for host inside property	10.00 %^a^	Instantaneous daily rate
Base mortality rate while resting inside property (non IRS)	10.00 %^a^	Instantaneous daily rate
Base mortality rate while outdoors and not searching	10.00 %^a^	Instantaneous daily rate
Base mortality when attempting to feed—pre bite	4.88 %^c^	Probability of death
Base mortality when attempting to feed—post bite	4.88 %^c^	Probability of death
Base mortality when attempting to oviposit—pre lay	0.00 %^b^	Probability of death
Base mortality when attempting to oviposit—post lay	0.00 %^b^	Probability of death
Probability vector deflected away from eave tube property	20.00 %^d^	Probability
Probability vector killed when attempting to enter eave tube property	70.00 % (*An. gambiae*)^e^ 52.00 % (*An. arabiensis*)^e^	Probability
Probability vector killed by eave tube when exiting eave tube property	0.00 %^f^	Probability
Probability vector deflected away from human under LLIN	60.00 %^g^	Probability
Probability vector killed by LLIN when attacking protected human	40.00 %^g^	Probability
Probability vector killed by LLIN after biting protected human	40.00 %^g^	
Probability vector exits non-eave tube property if deflected away from human under LLIN	50.00 %^h^	Probability
Probability vector exits eave tube property if deflected away from human under LLIN	0.00 %^c^	Probability
Probability deflected away from IRS protected property before entering	50.00 %^i^	Probability
Probability killed by IRS whilst resting in IRS treated property	40.00 %^i,j^	Probability

^a^Estimated from [19], Tables 2 and 3

^b^Conservative or neutral assumptions

^c^Estimated from [20]

^d^No definitive data available. A non-zero value was chosen and the full range of deflection values explored in Figs. 3 and 4

^e^Snetselaar et al. *pers. comm*

^f^Intentionally conservative estimate of exiting mortality

^g^Deflection and mortality were estimated as total mortality of 40 % based on [21] Tables 1 and 2, [22], Table 2d (44–47 % survival of fed mosquitoes with treated nets vs 93 % for controls, and 38 vs. 78 % of unfed), and [23] for reduced entry rate and slightly elevated exit rate, plus estimates of mortality in Tables 2 and 5

^h^[24] *Anopheles gambiae* house exiting rate

^i^Estimated from [25]

^j^Within range from [26] Tables 1 and 2, and [25]

The key metric generated by the model is the relative transmission potential (RTP). This is calculated as the number of infectious bites per (adult) vector lifetime as a proportion of that with no intervention. When the following two assumptions can be considered valid, RTP also represents the relative number of infectious bites per person per unit of time. The first assumption is that the juvenile population is at the carrying capacity of the available breeding sites and density dependence effects mean that any reduction in the populations’ rate of egg production arising from the interventions explored does not materially affect the recruitment rate of new adults to the vector population. When this assumption holds true, then the population age composition matches lifetime survival probabilities and the relative change in number of infectious bites per vector lifetime is equal to the relative change in bites from the vector population as a whole, per unit of time. The second assumption is that the human population size remains constant for different interventions. If this is true, then RTP is also equal to the proportion of infectious bites per person per unit of time under a given intervention compared to that with no intervention. Thus, for a vector population in which density dependence can be assumed to result in maintenance of a constant adult recruitment rate even when adult mortality is increased by interventions, with human population size unaffected by the intervention, the RTP should map directly to a proportionate change in the entomological inoculation rate (EIR). To illustrate, a 90 % RTP means a 10 % reduction in infectious bites per vector per lifetime and, subject to the assumptions above, represents a 10 % reduction in infectious bites from the vector population per unit of time and a 10 % reduction in infectious bites received per person per unit of time. Equivalently, 10 % RTP means the infectious bites per person per unit of time have been reduced by 90 %. This metric is calculated as an average across the human population and broken down into results for sub-groups with different interventions in place.

## Results

**Fig. 2:**
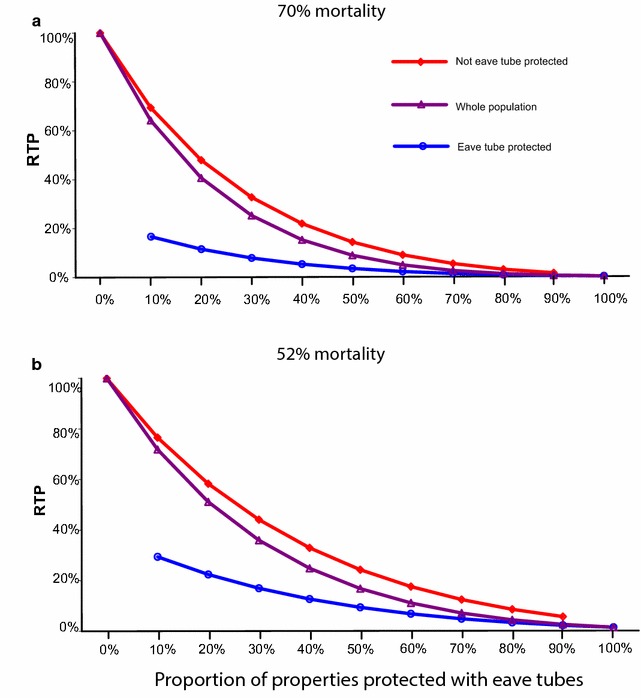
The effect of eave tubes on transmission potential of the vector population relative to no intervention (relative transmission potential, RTP). The* different lines* indicate different human host categories, with the *lowest line* (*blue*, least RTP) being the population protected by eave tubes, the* uppermost line* (*red*, greatest RTP) the population without any protection, and the* middle line* (*purple*) an overall RTP of the entire human host population. All plots use the same model assumptions: no other existing interventions, no mosquitoes are assumed killed on exit from an eave tube house, and 20 % deflection (without kill) by eaves tubes. Of those not deflected, mortality is estimated based on experimental data that used deltamethrin-treated eave tubes and showed either 70 % (**a**) of *An. gambiae*, or 52 % (**b**) of *An. arabiensis* were killed by the eave tubes (Snetselaar et al. *in prep*). Plots for eave tube-protected humans begin at 10 % coverage, as this is an empty category with 0 % eave tube coverage

Initial analyses consider the effect of the eave tubes technology (which as stated, includes house screening as described in [15]), assuming no other interventions are applied. Figure 2 summarizes results assuming eave tube coverage of between 0 and 100 %, showing the RTP experienced on average across the whole human population, and separately for the parts of the population in eave tube and non-eave tube properties. The results reveal a non-linear relationship between eave tube coverage and reduction in infectious bites, and show a community level effect, reducing infectious bites for people in unprotected as well as protected properties. For example, assuming eave tubes cause 70 % mortality (Fig. 2a), then if only 20 % of properties are protected by eave tubes, the properties without eave tubes still experience a reduction in RTP of >50 %. The RTP of eave tube protected houses continues to drop with greater coverage, and when only half the properties are outfitted with eave tubes, RTP is less than 20 % for the non-protected houses.

**Fig. 3:**
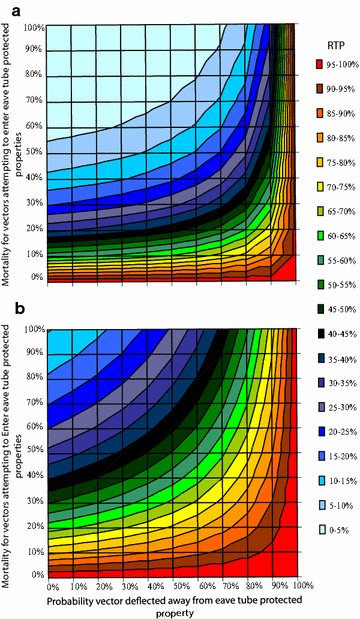
Effects of mosquito deflection and mortality on the impact of eave tubes on relative transmission potential (RTP). Plots show RTP across all combinations of deflection away from eave tube protected properties (*x* axis) and mortality for vectors attempting to enter (*y* axis). The* different colored contours* indicate different levels of RTP. **a**, **b** assume 70 and 30 % of properties are protected with eave tubes, respectively. No other interventions (LLINs or IRS) are assumed

Eave tubes impact vector survival and feeding in two ways; by deflecting mosquitoes away from eave tube protected properties and by killing mosquitoes that attempt to enter. Although deflection protects occupants of properties with eave tubes, it does little to reduce overall RTP in the absence of either high mortality or non-human host choice during outdoor searching, since deflected mosquitoes can locate and enter unprotected properties instead. From Fig. 3 it can be seen that overall RTP can be substantially reduced by installation of eave tubes, despite high deflection, given high enough eave-tube generated mortality and coverage. For the experimentally observed values of 52–70 % mortality (above that of controls with open eaves) (Snetselaar et al*. pers. comm.*) and an assumed 20 % deflection, RTP is reduced by more than 90 % with 70 % eave tube coverage (Fig. 3a) and by more than half with 30 % eave tube coverage (Fig. 3b).

**Fig. 4:**
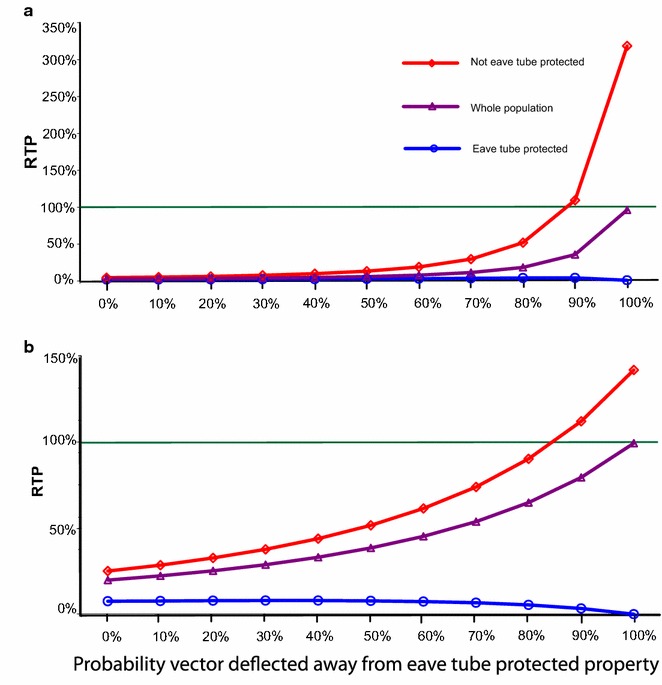
Effects of eave tube deflection and mortality assumptions on relative transmission potential (RTP) for people in houses with and without eave tubes. Plots show overall RTP across the population (*purple*,* middle line*), together with values for people with (*blue*,* lowest line*) and without (*red*,* uppermost line*) eave tube protection. Mortality for vectors attempting to enter eave tube protected property is assumed to be 70 % as a baseline, with 70 % (**a)** and 30 % (**b**) of properties protected by eave tubes. No LLINs or IRS are assumed. The *green lines* indicate the no-intervention value. Note the different vertical axis scales in **a** and **b**

When eave tube parameters are set to high deflection in combination with low mortality, people in eave tube properties remain protected, but mosquitoes can be redirected to unprotected properties. Figure 4 shows that, assuming eave tubes cause 70 % mortality, the average infectious bites experienced by the population overall are reduced irrespective of the probability of deflection, and this reduction is substantial for people in protected properties, with RTP kept close to zero. For all but the highest deflection values there is also some benefit to people in unprotected properties of reduced RTP. As illustrated in Fig. 4, with 70 % mortality, and 70 % (Fig. 4a) and 30 % (Fig. 4b) eave tube coverage, for deflection probabilities up to 80 % eave tubes still offer some benefit to unprotected people. Above this level, however, there is potential for the unprotected part of the population to experience increased infectious bites (plot crosses the green line). This effect is more severe with increasing coverage (Fig. 4a) as this causes increasing numbers of mosquitoes to be deflected to a diminishing pool of unprotected people, until coverage reaches 100 %, whereupon none of the population fall into the no eave tube category.

**Fig. 5:**
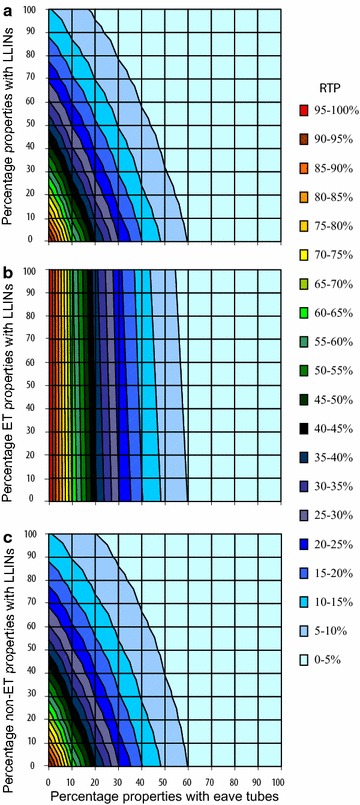
**a**–**c** Effect of altering coverage of households with eave tubes and LLINs on relative transmission potential (RTP). The *x* axis represents the percent of properties with eave tubes; the *y* axis represents the percent of properties with LLINs. It is assumed LLINs are used by 70 % of occupants in properties with LLINs. Eave tubes are assumed to cause 20 % deflection and 70 % mortality for remaining vectors attempting to enter an eave tube protected property (as described in Table 1). The distribution of each intervention differs among the plots. The* different colored contours* indicate different levels of RTP. **a** The distribution of each intervention is assumed random and not affected by the presence of the other intervention. **b** LLINS are present only in percentage of eave tube protected properties. **c** LLINS present only in percentage of non-eave tube protected properties

The analysis so far has considered the effects of eave tubes alone yet in reality, eave tubes will likely be introduced into areas where LLINs or IRS are already deployed. Figure 5 illustrates the combined effect of eave tubes and LLINs on RTP, depending on coverage of either intervention. For LLINs it is assumed that if properties have LLINs, 70 % of residents use them, reflecting real-world issues with achieving consistent levels of very high LLIN use, even when nets are available [27, 28]. LLINs are set to an overall 40 % mortality rate with 60 % deflection assuming that pyrethroids used on the LLINs cause excitorepellency (estimates consistent with mortality and deflection levels in experimental hut trials conducted in Côte d’Ivoire and Benin [21, 23, 29]). Impact of eaves tubes follows the baseline assumptions of 70 % mortality and 20 % deflection. It is assumed that the interventions themselves are allocated randomly among properties, with assumptions of independence of each intervention further explored. It can be seen from Fig. 5a–c that both eave tubes and LLINs can offer incremental benefits in contexts where the other intervention is already in use. This can be seen by considering horizontal or vertical transects through the plot, representing a constant value for LLINs (horizontal) or eave tube (vertical) usage. Where the scale is sufficiently fine-grained, it can be seen that RTP reduces with increasing coverage of the other intervention.

In Fig. 5a–c the allocation of interventions between properties varies, considering the extreme scenarios of completely overlapping (Fig. 5b, i.e. interventions are always deployed together) and complementary (Fig. 5c, LLINs are specifically targeted to houses without eave tubes) deployment strategies, as well as a random allocation (Fig. 5a). These figures show that benefits of adding LLINs exclusively to properties which are already protected with eave-tubes are only marginal. For example, in Fig. 5b where interventions are completely overlapping (i.e. deployed together in the same property), if 60 % of properties have eave tubes then adding LLINs to even 100 % LLINs provides only about a 5 % additional reduction in RTP. However, using LLINs only in properties which have no eave tube protection, as in Fig. 5c, gives benefits comparable to those achieved when all properties have LLINs, and better reduction in RTP for all properties overall.

**Fig. 6:**
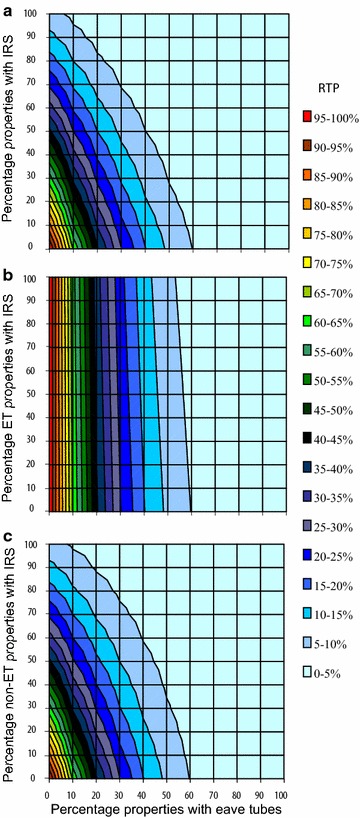
**a**–**c** Effect of altering coverage of households with eave tubes and IRS on relative transmission potential (RTP). The *x* axis represents the percent of properties with eave tubes; the *y* axis represents the percent of properties with IRS. IRS is assumed to deflect 50 % of vectors that attempt to enter a property, and to kill 40 % of resting vectors as in Table 1. Eave tubes are assumed to deflect 20 % of vectors and cause 70 % mortality in the remainder attempting to enter an eave tube protected property. The distribution of each intervention differs among the plots. The* different coloured contours* indicate different levels of RTP. **a** The distribution of each intervention is assumed random and not affected by the presence of the other intervention. **b** LLINS are present only in percentage of eave tube protected properties. **c** LLINS present only in percentage of non-eave tube protected properties

Figure 6a–c shows the effect of combining eave tubes with IRS, assuming each intervention is applied randomly across the population without regard for whether the other intervention (or any) is already in place (Fig. 6a). It can be seen that increasing eave tube coverage has a greater impact on RTP than increasing IRS coverage. Model assumptions for eave tubes are again, 20 % deflection and 70 % mortality for those not deflected. Mortality from IRS is set to 40 % (with 50 % deflection), which is in line with certain empirical data [26]. Note that if IRS kill is set to 70 %, then IRS performs nearly as well as eave tubes (Additional file 2, Sensitivity analysis). As with LLINs described in Figs. 5b, 6b shows that adding IRS to the same properties that have eave tubes (i.e. redundant distribution) offers limited additional reduction in infectious bites. However, applying IRS to the properties that do not have eave tubes (Fig. 6c, i.e. complementary distribution) offers substantial benefits, reducing overall RTP across the parameter space.

## Discussion

The modelling study reveals that eave tubes could reduce the number of infectious bites a malaria mosquito will transmit in a population, and from Fig. 2, it appears that the impact of eave tubes could be substantial even with low proportions of properties outfitted with this intervention. With only 50 % eave tube coverage, the average infectious bites per vector lifetime, per person, are reduced by more than 80–90 % for the whole human population. The benefit is greater for those who are in the houses to which eaves tubes have been fitted, but even those in houses without screening and eave tubes gain substantial community benefit.

As with all models, the outputs in the current study depend on the assumptions. The baseline parameters provided in Table 1 were selected as representative of the available literature. Sensitivity analysis (Additional file 2) demonstrates that although variation in different parameters can affect the quantitative results, the non-linear reduction in relative transmission potential with increasing coverage appears robust, indicating a mass action effect, similar to that observed with LLINs. This is an important finding because it suggests that there should be community benefits in locations where only a modest proportion of houses receive eave tubes (either because of poor adoption or because properties are not amenable to have tubes fitted).

Both deflection and reduced kill are predicted to degrade eave tube efficacy (Fig. 3). Greater deflection means that fewer mosquitoes encounter the active and if they aren’t killed, then the combined effects could make the eave tubes much less effective. Yet it is worth noting that basic house screening without the addition of insecticide, which would be represented in the current model as 100 % deflection without kill, has been shown to reduce malaria transmission in multiple studies [9, 30–32]. Furthermore, one study in the Gambia demonstrated explicitly that unscreened houses adjacent to screened houses did not suffer increased disease burden due to deflection of mosquitoes [9]. These empirical data suggest that the model outputs are likely conservative with respect to overall impact since any level of killing should improve control relative to screening alone. Also any non-human host feeding, such as on livestock, is not captured in the model, which could further dilute malaria transmission [33]. Nonetheless, the model reveals the potential importance of having an effective active ingredient within the tubes and supports the need for regular retreatment or replacement of the electrostatic netting to ensure the killing effect is maintained and any risks of deflection are minimized.

The model results for combining eave tubes with existing interventions demonstrate benefits of developing integrated strategies, although this depends crucially on how the interventions are deployed with respect to one another. Under the baseline assumptions, eave tubes perform better than either LLINs or IRS for a given level of coverage. If eave tubes are fitted to the exact same houses as receive IRS or LLINs, there is potential for marked redundancy between technologies (Figs. 5b, 6b). However, with random distribution (Figs. 5a, 6a), or better still strategic distribution wherein overlap in interventions is minimized (Figs. 5c, 6c), there is greater complementarity. This result is important in terms of optimizing interventions on a per house basis. Not all houses within a location will necessarily be amenable to installation of eave tubes (either because of the physical nature of the house or perhaps user acceptance). Targeting these houses with IRS, or ensuring the occupants have full access to LLINs, would maximize control. Likewise, compliance with LLINs or IRS can sometimes be very low (refusal rates for IRS can be as high as 70 % for example [34]). These households could provide primary targets for installation of eave tubes.

## Conclusions

Overall, the results of the modelling suggest that the eave tube technology could affect malaria incidence by reducing the number of infectious bites from mosquitoes. Individual householders should gain immediate personal protection, as well as relief from nuisance mosquitoes, which should encourage adoption. As coverage increases, mass action effects should yield additional community-wide benefits. There also appears potential for integration with existing interventions. These results support the further research and development of the eave tube technology.

## Additional files

10.1186/s12936-016-1505-1 Eave tubes simple model.
10.1186/s12936-016-1505-1 Sensitivity analysis.

## Abbreviations

IRS: indoor residual spraying
LLIN: long-lasting insecticide-treated bed net
RCT: randomized controlled trial
RTP: relative transmission potential
EIR: entomological inoculation rate
