# Supplementary material for: Eave tubes for malaria control in Africa: a modelling assessment of potential impact on transmission
Source: Malar J. 2016 Sep 2;15(1):449. doi: 10.1186/s12936-016-1505-1 (PMC5009529; doi:10.1186/s12936-016-1505-1)
Supplement: Supplementary file 1 — 10.1186/s12936-016-1505-1 Eave tubes simple model. [file 12936_2016_1505_MOESM1_ESM.pdf]

Eave tubes simple model

t_1 average search time to locate a property
t_2 average search time to locate a human host when searching indoors
t_3 average time spent resting indoors post-feed
t_4 average time spent finding ovipositing site
t_5 average time spent from ovipositing to host searching
t_6 cycles from infection to infectiousness
μ_1 base mortality rate whilst searching for property or laying site
μ_2 base mortality rate whilst searching for host inside property
μ_3 base mortality whilst resting inside property (non IRS)
μ_4 base mortality rate whilst outdoors and not searching
P_1 proportion of properties with external protection (eave tubes)
P_2 proportion of properties with eave tubes protected with IRS
P_3 proportion of properties without ET which are protected with IRS
P_4 proportion of ET properties protected with bednets
P_5 proportion of people under bednets in ET properties with bednets
P_6 proportion of non-Et properties protected with bednets
P_7 proportion of people under bednets in non-ET properties with bednets
P_8 probability per feed that mosquito will acquire Plasmodium infection
P_9 probability that an entered ET property will have IRS
P_{10} probability that an entered non-ET property will have IRS
D_1 probability mosquito deflected away from ET-protected house
D_2 probability deflected away from human under ITN
D_3 probability exits ET property if deflected away from net-protected human
D_4 probability exits non-ET property if deflected away from net-protected human
D_5 probability deflected away from IRS protected property before selecting a host
M_1 if not deflected, probability mosquito killed before entering ET protected house
M_2 probability mosquito killed by ET/eave nets etc when exiting protected house
M_3 if not deflected, prob mos. killed by bed net before feeds on human under net
M_4 probability killed by bed net after feeding on human under net
M_5 base mortality when attempting to feed - pre bite
M_6 base mortality when attempting to feed - post bite
M_7 base mortality when attempting to oviposit - pre lay
M_8 base mortality when attempting to oviposit - post lay
M_{10} probability killed by IRS whilst resting in IRS treated property

Probability survives search time to locate a property $S_1 = e^{-t_1\mu_1}$

Probability survives search to locate a human host indoors $S_4 = e^{-t_2\mu_2}$

Total probability deflected from located ET property without attempting to feed

$$D_6 = D_1 + (1-D_1)(1-M_1)\left(P_2D_5 + (1-P_2D_5)(1-M_2)\frac{P_4P_5S_4D_2D_3}{1-S_4P_5D_2(1-D_3)}\right)$$

Total probability exits non-ET property without attempting to feed

$$D_7 = P_3D_5 + (1-P_3D_5)P_6\frac{S_4P_7D_2D_4}{1-S_4P_7D_2(1-D_4)}$$

Probability survives searching & enters a no-ET property $S_2 = S_1(1-P_1)(1-P_3D_5)$

Probability survives searching and enters an ET property $S_3 =$
$S_1P_1(1-D_1)(1-P_2D_5)(1-M_1)$

Probability survives background mortality whilst resting indoors $S_5 = e^{-t_3\mu_3}$

Probability survives background mortality whilst finding laying-site $S_6 =$
$e^{-t_4\mu_1}(1-M_7)$

Probability survives background mortality between laying and host searching
$S_7 = e^{-t_5\mu_4}(1-M_8)$

Probability survives & attempts to feed on human under bed net (in ET property)

$$S_8 = \frac{P_4S_4P_5(1-D_2)}{(1-S_4P_5D_2(1-D_3))}$$

Probability survives & attempts to feed on human without bed net (in ET property)

$$S_9 = S_4\left(1-P_4+\frac{P_4(1-P_5)}{1-S_4P_5D_2(1-D_3)}\right)$$

Probability survives attacking to bite if feeding on bed-net protected human (in ET property) $S_{10} = (1-M_3)(1-M_5)$

Probability survives attacking to bite if feeding on human without bed net (in ET property) $S_{11} = 1-M_5$

Probability survives feeding post-bite if feeding on bed-net protected human (in ET property) $S_{12} = (1-M_4)(1-M_6)$

Probability survives feeding post bite if feeding on human without bed net (in ET property) $S_{13} = 1-M_6$

Probability survives indoor search and bites human under bed net (in ET property)
$S_{14} = S_8S_{10}$

Probability survives indoor search and bites human without bed net (in ET property)

$S_{15} = S_9 S_{11}$

Probability survives locating and biting human under bed net (in ET property)

$S_{16} = S_{14} S_{12}$

Probability survives locating and biting human without bed net (in ET property)

$S_{17} = S_{15} S_{13}$

Probability vector in ET property is in IRS treated property

$$P_9 = \frac{P_2(1-D_5)}{P_2(1-D_5)+(1-P_2)}$$

Probability survives finding host, feeding, resting and exiting property in IRS treated property (in ET property) $S_{18} = (S_{16}+S_{17})S_5 P_9(1-M_{10})(1-M_2)$

Probability survives finding host, feeding, resting and exiting property in unsprayed property (in ET property) $S_{19} = (S_{16}+S_{17})S_5(1-P_9)(1-M_2)$

Probability survives & attempts to feed on human under bed net (in non-ET property)

$$S_{20} = \frac{S_4 P_6 P_7(1-D_2)}{\left(1-S_4 P_7 D_2(1-D_4)\right)}$$

Probability survives & attempts to feed on human without bed net (in non-ET property) $S_{21} = S_4\left(1-P_6+\frac{P_6(1-P_7)}{1-S_4 P_7 D_2(1-D_4)}\right)$

Probability survives attacking to bite if feeding on bed-net protected human (in non-ET property) $S_{22} = (1-M_3)(1-M_5)$

Probability survives attacking to bite if feeding on human without bed net (in non-ET property) $S_{23} = 1-M_5$

Probability survives feeding post-bite if feeding on bed-net protected human (in non-ET property) $S_{24} = (1-M_4)(1-M_6)$

Probability survives feeding post bite if feeding on human without bed net (in non-ET property) $S_{25} = 1-M_6$

Probability survives indoor search and bites human under bed net (in non-ET property) $S_{26} = S_{20} S_{22}$

Probability survives indoor search and bites human without bed net (in non-ET property) $S_{27} = S_{21} S_{23}$

Probability survives locating and biting human under bed net (in non-ET property)

$S_{28} = S_{24} S_{26}$

Probability survives locating and biting human without bed net (in non-ET property)

$S_{29} = S_{25} S_{27}$

Probability vector in ET property is in IRS treated property

$$P_{10} = \frac{P_3(1-D_5)}{P_3(1-D_5)+(1-P_3)}$$

Probability survives finding host, feeding, resting and exiting property in IRS treated property (in non-ET property) $S_{30} = (S_{28}+S_{29})S_5 P_{10}(1-M_{10})$

Probability survives finding host, feeding, resting and exiting property in unsprayed property (in non-ET property) $S_{31} = \left(S_{28} + S_{29}\right) S_5 \left(1 - P_{10}\right)$

Overall Probability per cycle mosquito survives to bite

$$S_{32} = \frac{S_3\left(S_{14} + S_{15}\right) + S_2\left(S_{26} + S_{27}\right)}{\left(1 - S_1\left(D_6 P_1 + \left(1 - P_1\right) D_7\right)\right)}$$

Overall Probability per cycle mosquito survives to lay eggs

$$S_{33} = \frac{S_6\left(S_3\left(S_{18} + S_{19}\right) + S_2\left(S_{30} + S_{31}\right)\right)}{1 - S_1\left(D_6 P_1 + D_7\left(1 - P_1\right)\right)}$$

Overall Probability mosquito survives full cycle $S_{34} = S_{33} S_7$

Average infectious bites per vector per lifetime $LAIB = \dfrac{S_{32} P_8 S_{34}^{t_6}}{\left(1 - S_{34}\right)\left(1 - S_{34}\left(1 - P_8\right)\right)}$

$LAIB_0$ = average infectious bites per vector per lifetime with no intervention

Average infectious bites per vector per lifetime as proportion of value with no intervention $VLAIB = \dfrac{LAIB}{LAIB_0}$

The relative transmission potential (*RTP*) is defined as the average number of infectious bites per vector per lifetime per person expressed as a proportion of the number with no intervention

If we assume that juvenile density-dependence effects mean that the rate at which new adult vectors join the population is the same with and without intervention, and that the size of the human population is unaffected by any intervention, then *RTP* = *VLAIB*.

Average infectious bites per vector per lifetime per person for people using bednet in ET property

$$RTP_1 = RTP \times \frac{\text{proportion of bites per cycle taken on humans with ET \& net}}{\text{proportion of humans protected with ET \& net}}$$

$$= \frac{LAIB}{LAIB_0} \frac{S_3 S_{14}}{S_3\left(S_{14} + S_{15}\right) + S_2\left(S_{26} + S_{27}\right)} \frac{1}{P_1 P_4 P_5}$$

Average infectious bites per vector per lifetime per person for people not using bednet in ET property

$$RTP_2 = RTP \times \frac{\text{proportion of bites per cycle taken on humans with ET no net}}{\text{proportion of humans protected with ET \& no net}}$$

$$= \frac{LAIB}{LAIB_0} \frac{S_3 S_{15}}{S_3 \left(S_{14} + S_{15}\right) + S_2 \left(S_{26} + S_{27}\right)} \frac{1}{P_1 \left(1 - P_4 P_5\right)}$$

Average infectious bites per vector per lifetime per person for people using a bednet in no ET property

$$RTP_3 = RTP \times \frac{\text{proportion of bites per cycle taken on humans with net \& no ET}}{\text{proportion of humans protected with net \& no ET}}$$

$$= \frac{LAIB}{LAIB_0} \frac{S_2 S_{26}}{S_3 \left(S_{14} + S_{15}\right) + S_2 \left(S_{26} + S_{27}\right)} \frac{1}{\left(1 - P_1\right) P_6 P_7}$$

Average infectious bites per vector per lifetime per person for people not using bednet in no ET property

$$RTP_4 = RTP \times \frac{\text{proportion of bites per cycle taken on humans with no net \& no ET}}{\text{proportion of humans with no net \& no ET}}$$

$$= \frac{LAIB}{LAIB_0} \frac{S_2 S_{27}}{S_3 \left(S_{14} + S_{15}\right) + S_2 \left(S_{26} + S_{27}\right)} \frac{1}{\left(1 - P_1\right)\left(1 - P_6 P_7\right)}$$
