# Supplementary material for: Eave tubes for malaria control in Africa: a modelling assessment of potential impact on transmission
Source: Malar J. 2016 Sep 2;15(1):449. doi: 10.1186/s12936-016-1505-1 (PMC5009529; doi:10.1186/s12936-016-1505-1)
Supplement: Supplementary file 2 — 10.1186/s12936-016-1505-1 Sensitivity analysis. [file 12936_2016_1505_MOESM2_ESM.pdf]

Sensitivity analysis to support eave tube paper

Table of baseline parameter values used by model unless otherwise indicated

Description	value	units
Assumed Cycle length	3.00	*days*
Average search time to locate a property	0.50	*hours*
Search time to locate a human host when searching indoors	0.25	*hours*
Average time spent resting indoors post-feed	8.00	*hours*
Average time spent finding ovipositing site	8.00	*hours*
Average time spent from ovipositing to host searching	55.25	*hours*
Base mortality rate whilst searching for property or laying site	10.00%	*instantaneous daily rate*
Base mortality rate whilst searching for host inside property	10.00%	*instantaneous daily rate*
Base mortality rate whilst resting inside property (non IRS)	10.00%	*instantaneous daily rate*
Base mortality rate whilst outdoors and not searching	10.00%	*instantaneous daily rate*
Base mortality when attempting to feed - pre bite	4.88%	*probability of death*
Base mortality when attempting to feed - post bite	4.88%	*probability of death*
Base mortality when attempting to oviposit- pre lay	0.00%	*probability of death*
Base mortality when attempting to oviposit - post lay	0.00%	*probability of death*
Probability vector deflected away from ET property	20.0%	*probability*
Probability vector killed when attempting to enter ET property	70.0% (*An. gambiae*) [1] 52.0% (*An. arabiensis*) [1]	*probability*
Probability vector killed by ET when exiting ET property	0.0%	*probability*
Probability vector deflected away from human under LLIN	60.0%	*probability*
Probability vector killed by LLIN when attacking protected human	40.0%	*probability*
Probability vector killed by LLIN after biting protected human	40%	
Probability vector exits ET property if deflected away from human under LLIN	50.0%	*probability*
Probability vector exits non-ET property if deflected away from human under LLIN	0.0%	*probability*
Probability deflected from IRS property before attempting to feed	50.0%	*probability*
Probability killed by IRS when resting in IRS treated property	40.0%	*probability*

Cycle length

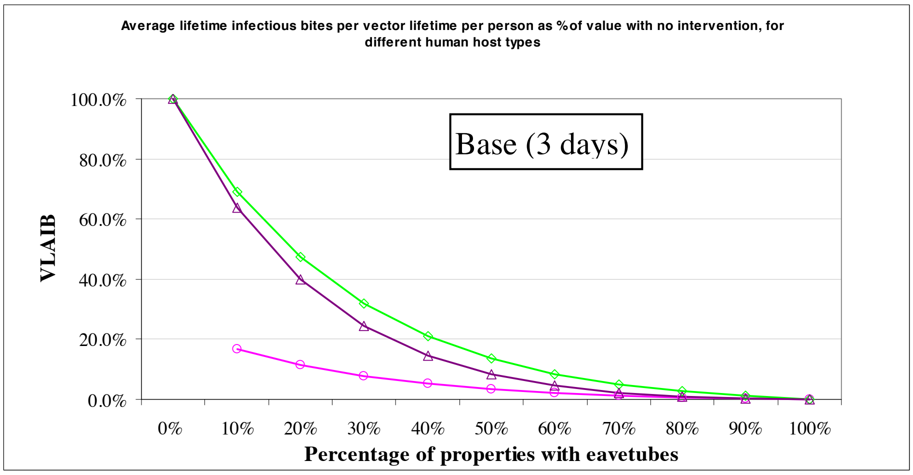

LAIB$_0$ = 0.0971

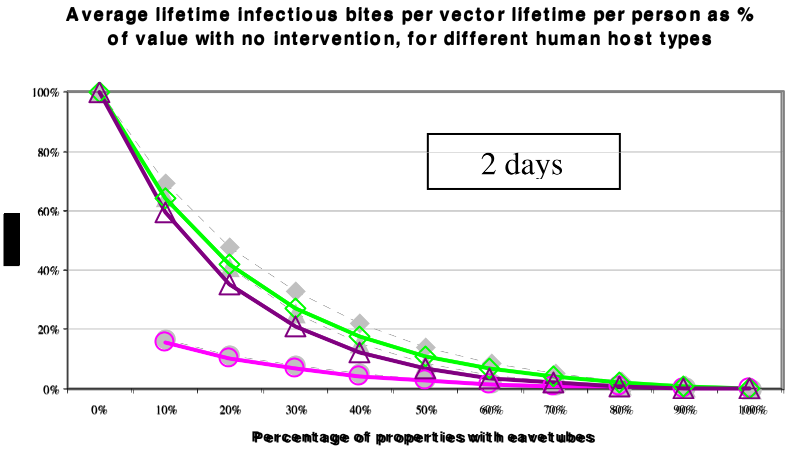

LAIB$_0$ = 0.2059

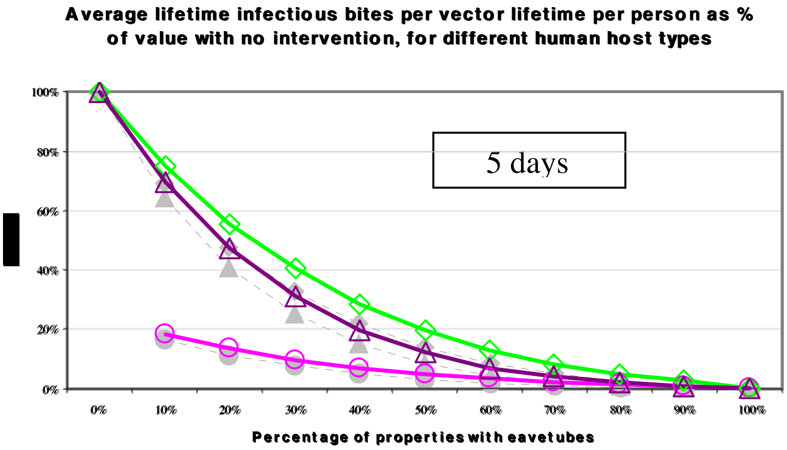

LAIB$_0$ = 0.0294

The LAIB$_0$ changes intuitively, increasing with a shorter cycle length and reducing with an increased cycle length. The VLAIB, the proportionate impact of the intervention, shows the reverse outcome, because the mortality added by the intervention is a larger proportion of the per cycle mortality with a short cycle length than with a longer cycle length.

Time to locate a property

Base = 0.5 hours

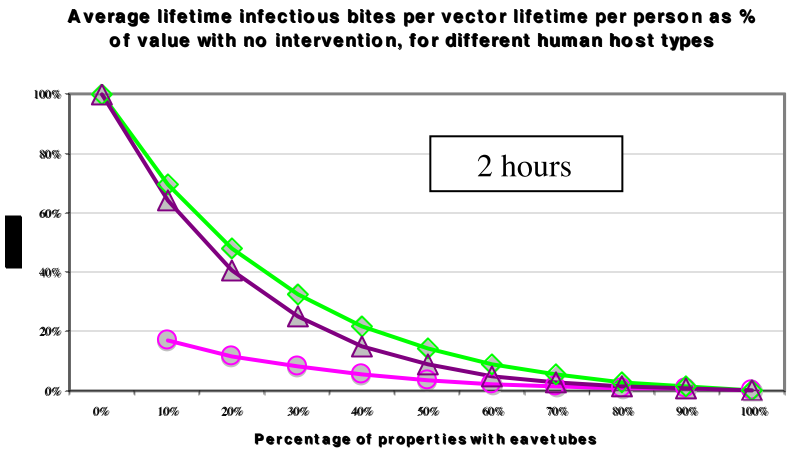

LAIB$_0$
= 0.0965

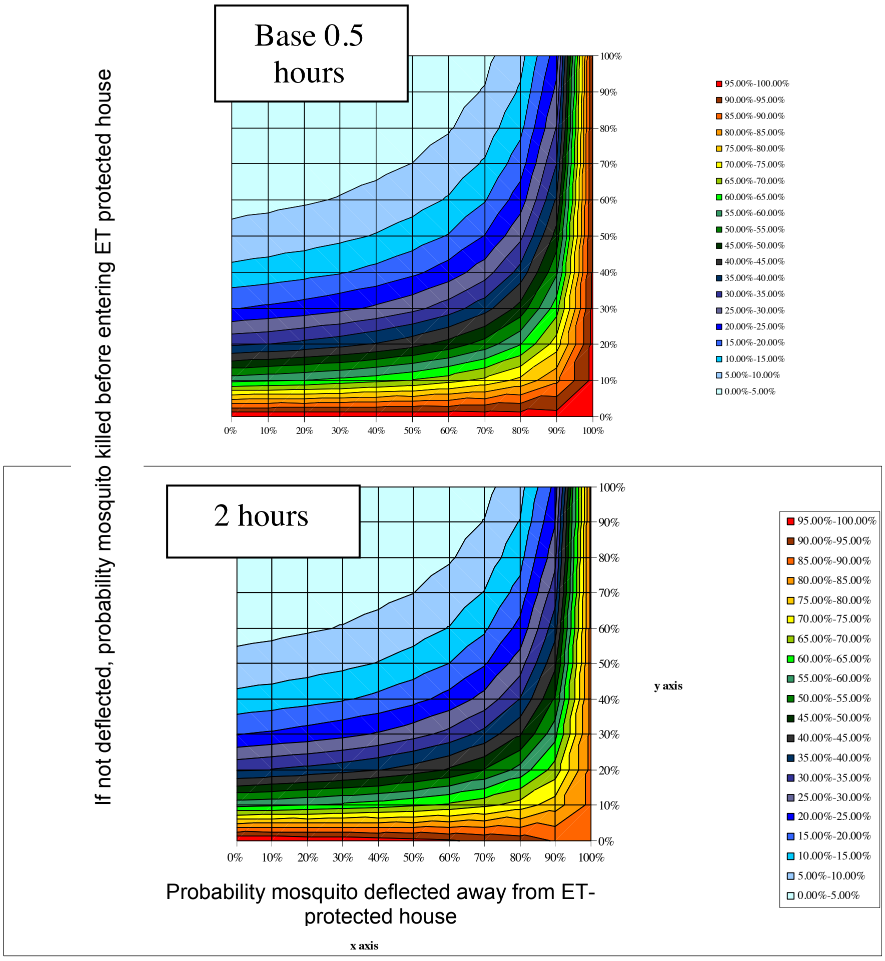

70% ET coverage

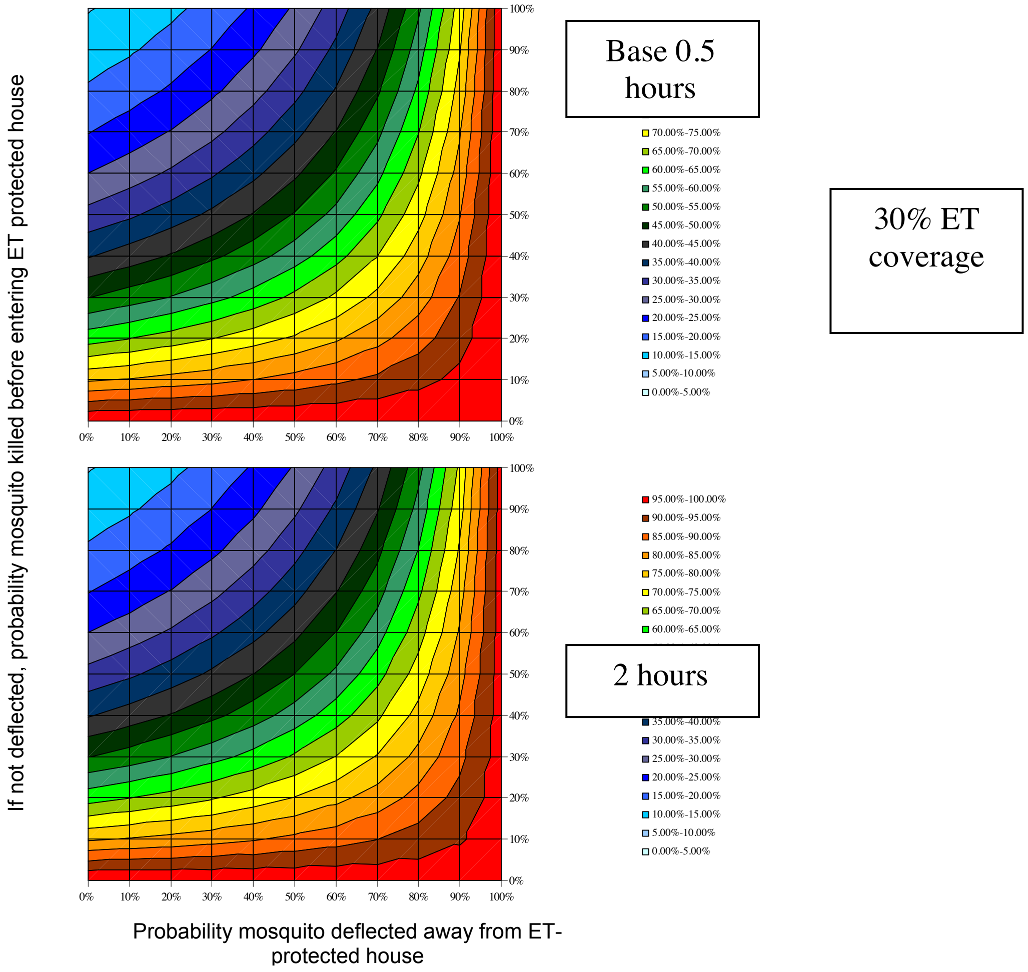

Extra time to locate property moves some mortality from post bite to pre-bite. Minimal effect on VLAIB for 0% probability of deflection, but increased mortality when locating a property means that the proportionate effect on LAIB of deflection away from properties is increased. If, however, vectors are killed by eave tubes before biting, then the additional mortality incurred by individuals which, had they survived to locate a property, would have been killed pre bite by the effect of eave tubes, is irrelevant, so the impact of an increased search time reduces with high ET mortality and coverage.

Time to locate a host indoors

Average infectious bites per vector lifetime as % no-treatment value for combinations of % properties with bed nets and % people under nets in those properties

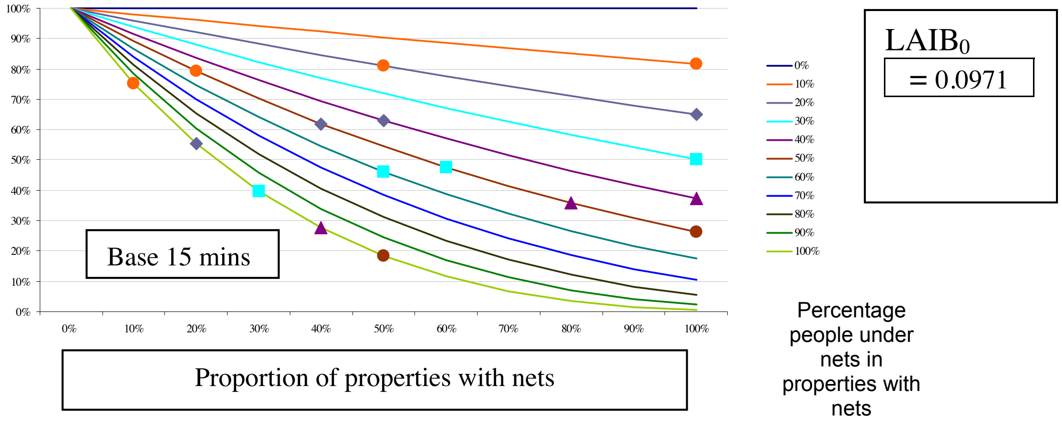

Average infectious bites per vector lifetime as % no-treatment value for combinations of % properties with bed nets and % people under nets in those properties

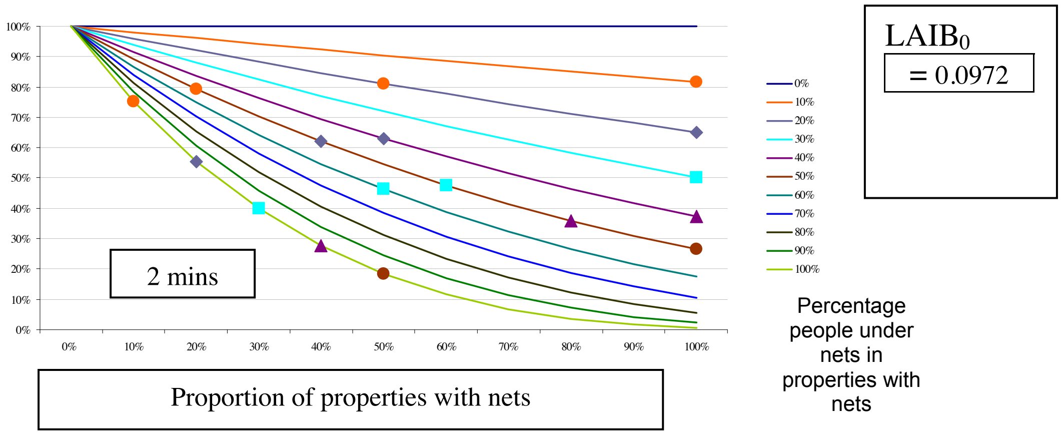

Average infectious bites per vector lifetime as % no-treatment value for combinations of % properties with bed nets and % people under nets in those properties

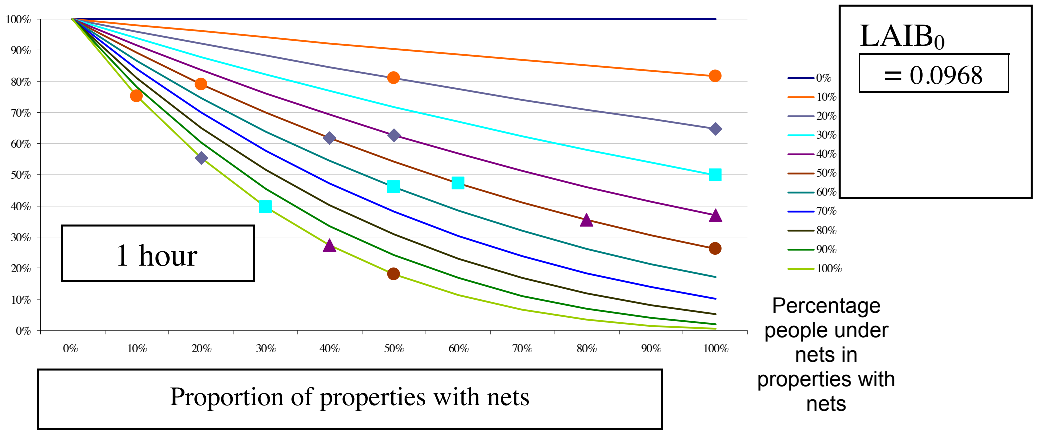

Minimal effect on $LAIB_0$ and VLAIB

Probability exits if deflected from net

Base = 50%

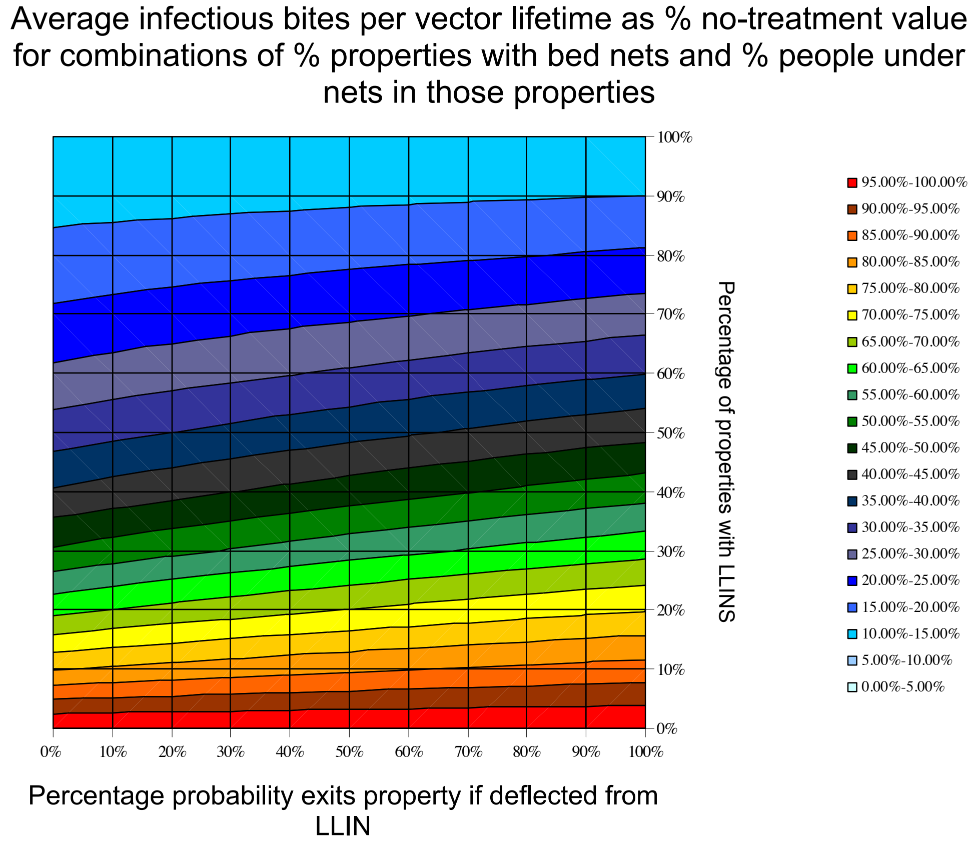

If exits bed net property, has some probability of entering non-bed net property, so higher exit gives higher infectious bites, as seen in this graph.

Average infectious bites per vector lifetime as % no-treatment value for combinations of % properties with bed nets and % people under nets in those properties

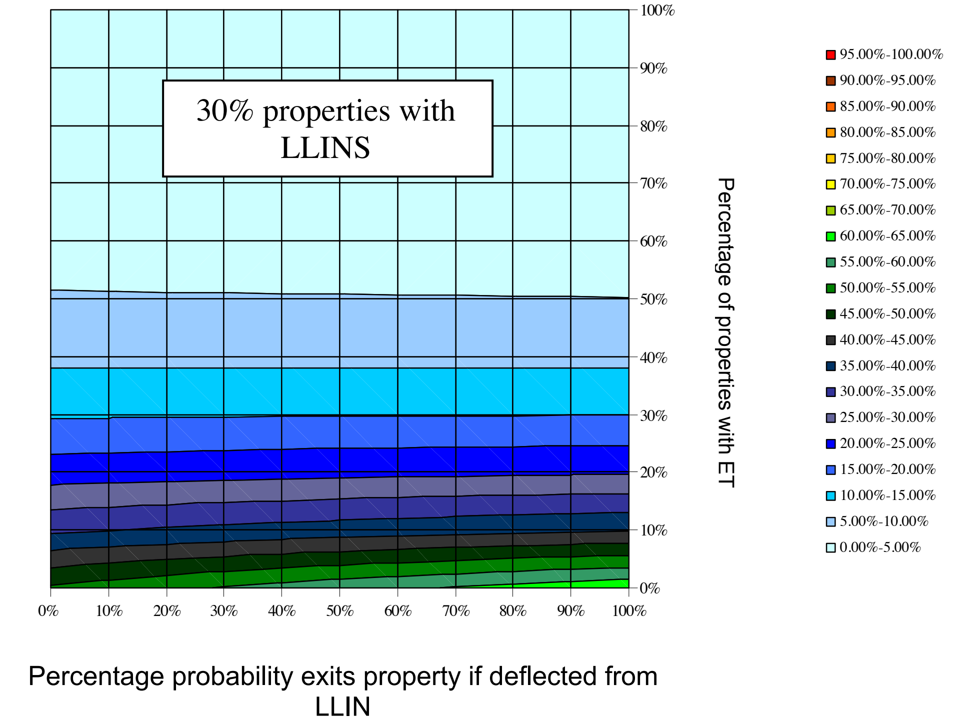

Exiting a bed net protected property may result in entering a non-protected property (higher infectious bites) or an ET protected property (lower infectious bites), so increasing prob of exit may make things worse until ET protection reaches high enough level, then increasing exit will make things better, as seen in this graph.

Average infectious bites per vector lifetime as % no-treatment value for combinations of % properties with bed nets and % people under nets in those properties

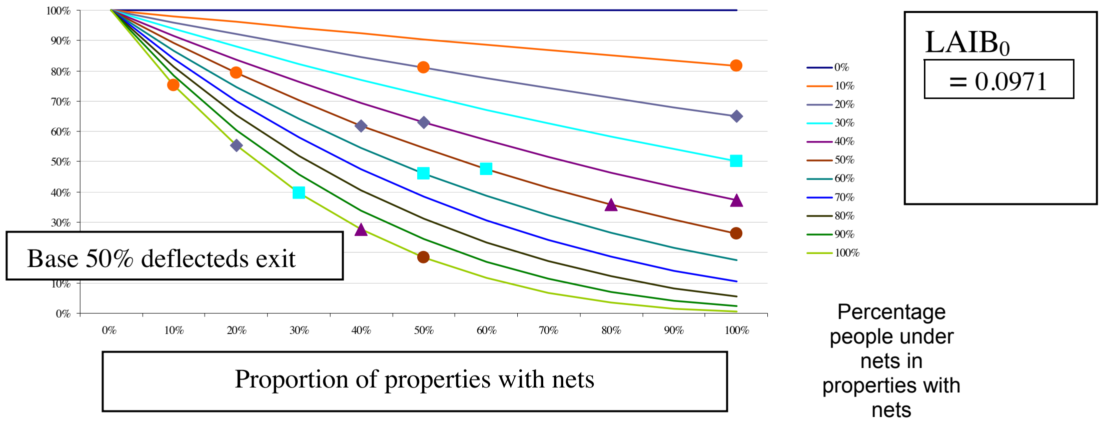

Average infectious bites per vector lifetime as % no-treatment value for combinations of % properties with bed nets and % people under nets in those properties

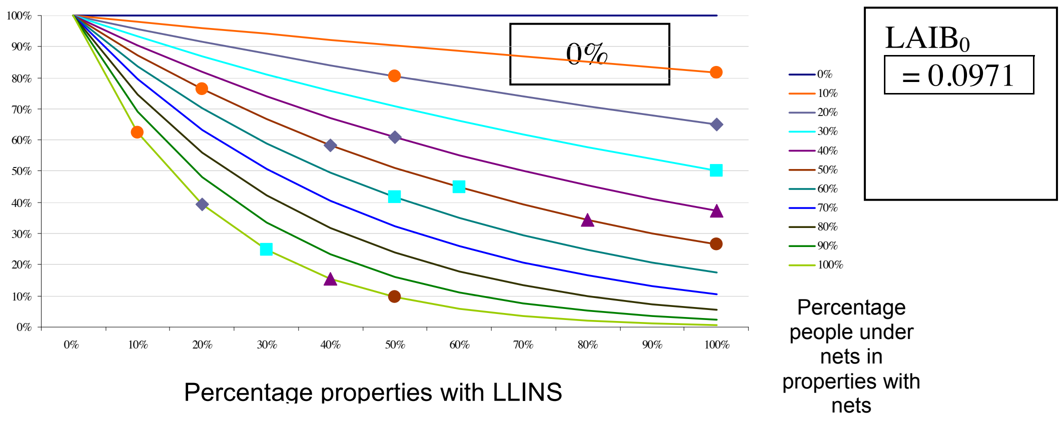

Average infectious bites per vector lifetime as % no-treatment value for combinations of % properties with bed nets and % people under nets in those properties

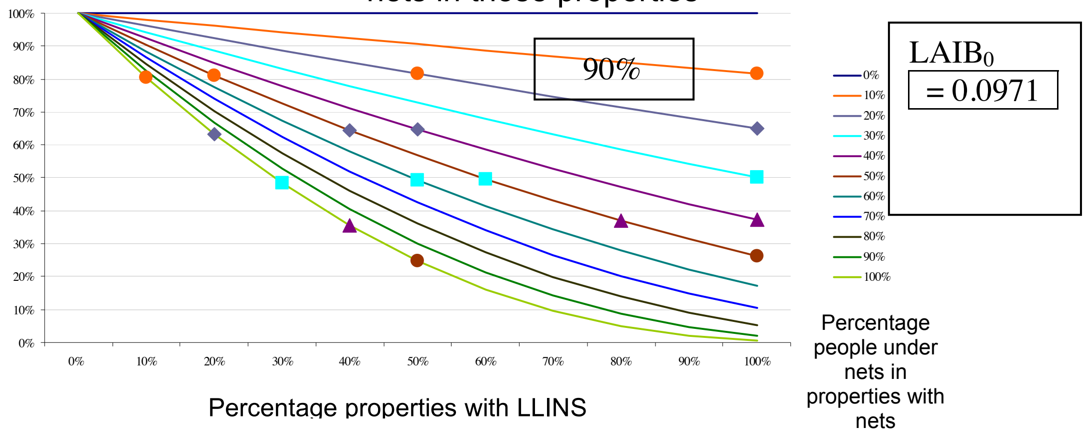

Higher exit probabilities diminishes the differential between property coverage and bednet coverage within properties, but does not reverse it. At 100% exit, there is no difference in VLAIB for clustered or disbursed bed net use.

Background Instantaneous Mortality Rate

Base = 10% per day

Average infectious bites per vector lifetime per person as % of value with no intervention, for different human host types

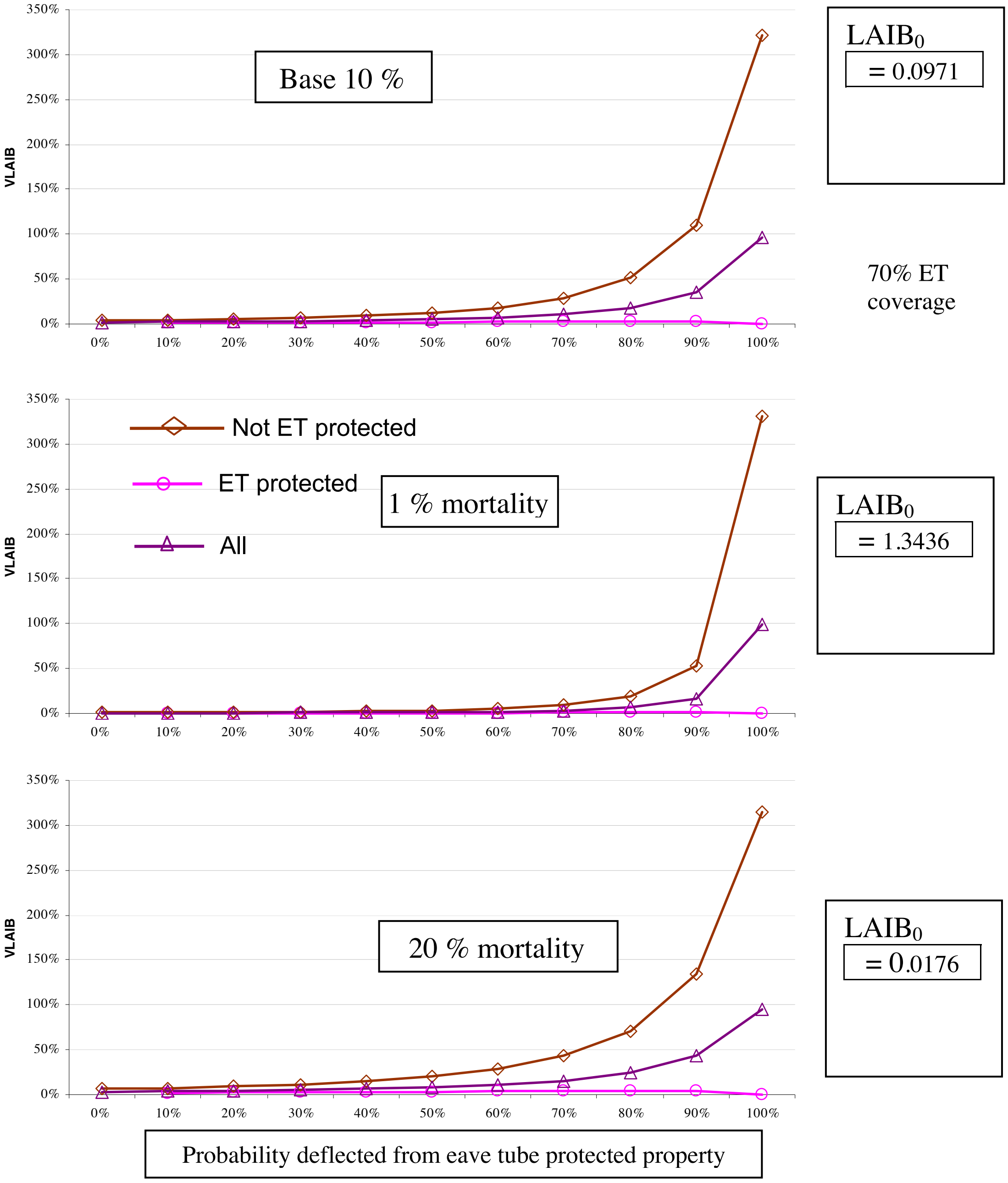

Average lifetime infectious bites per vector lifetime per person as % of value with no intervention, for humans with various protection types

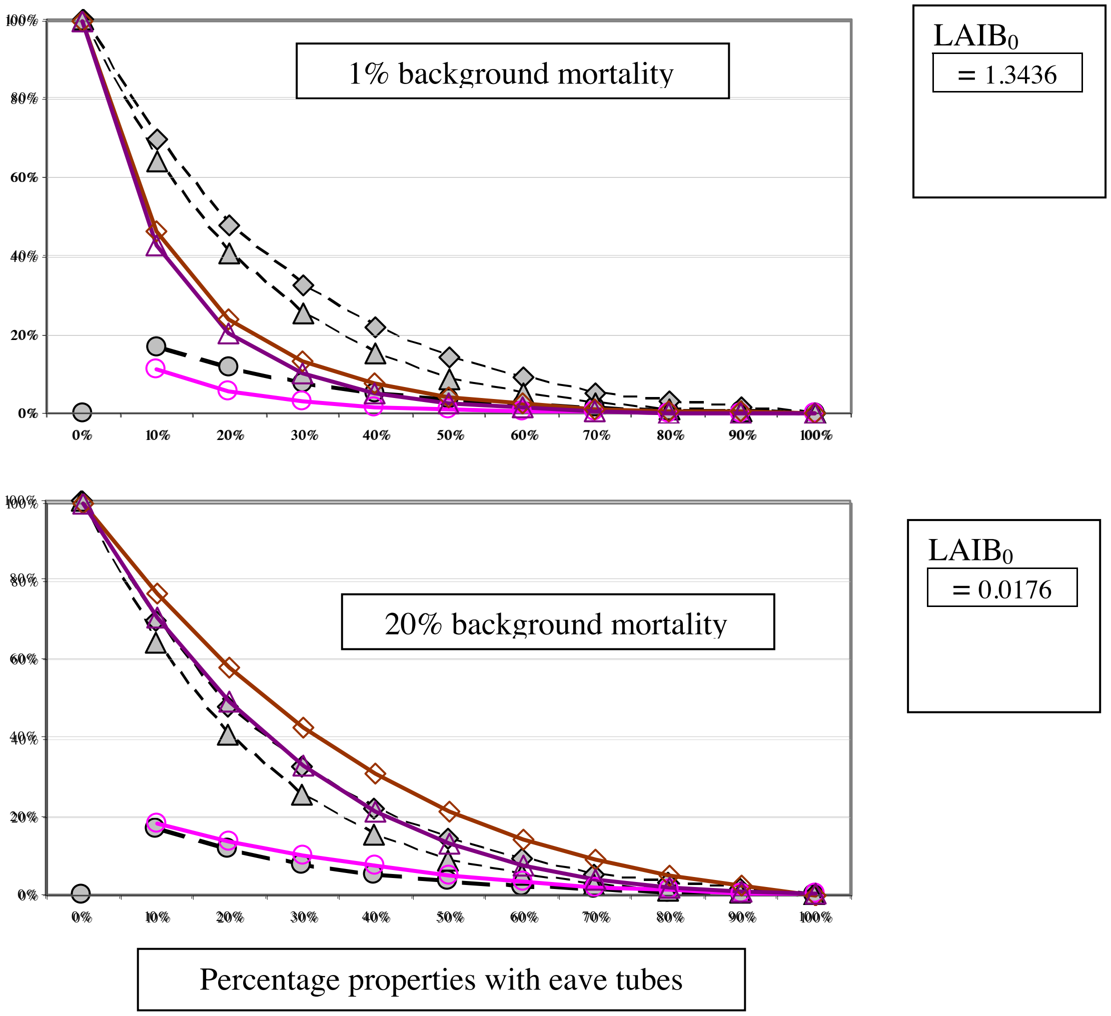

With higher background mortality the base $LAIB_0$ is reduced, but the proportionate impact of the mortality caused by interventions is less.

Average lifetime infectious bites per vector lifetime per person as % of value with no intervention, for different ET and LLIN coverage

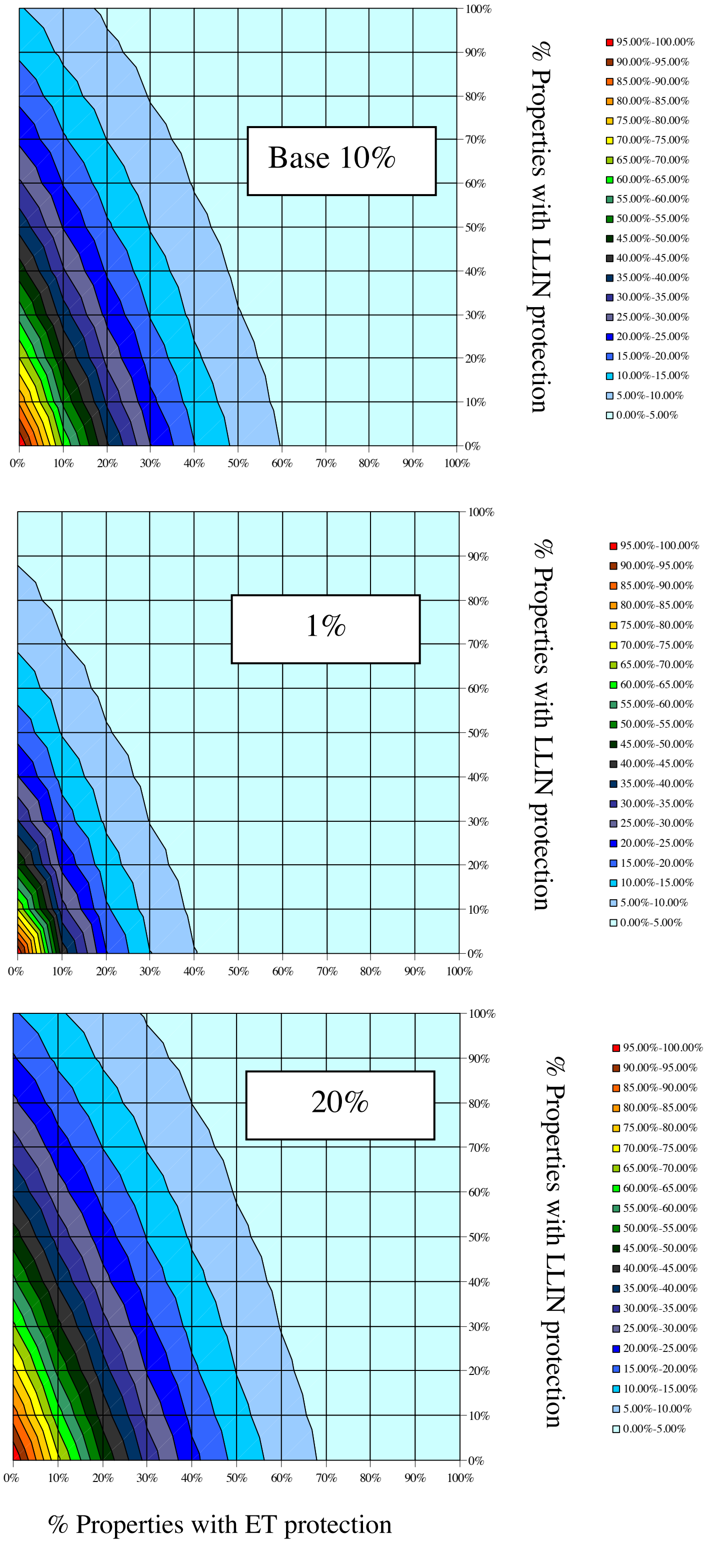

Background Feeding-related mortality (pre & post bite)

Base = 4.9% pre bite and 4.9% post bite

Average lifetime infectious bites per vector lifetime per person as % of value with no intervention, for different ET and LLIN coverage

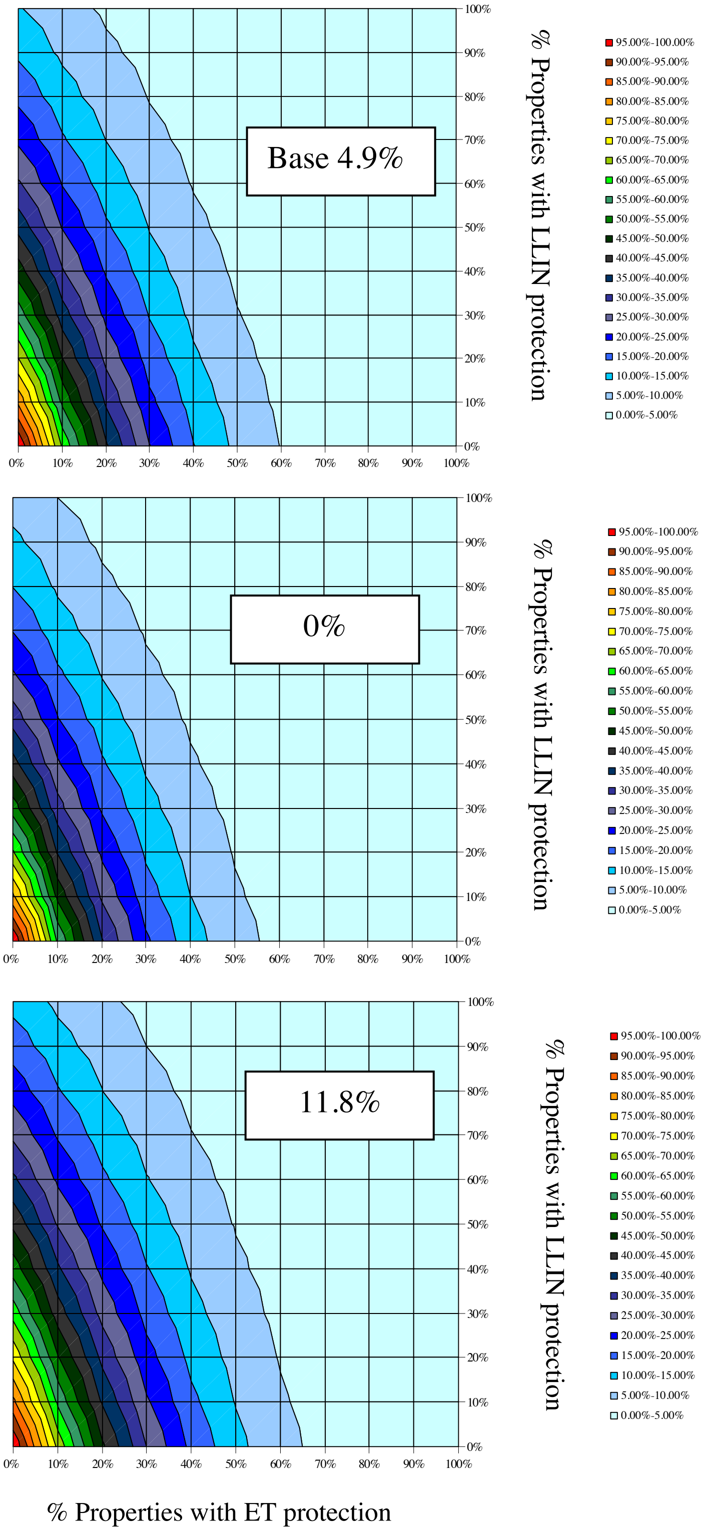

4.9% = 10% per feed, 11.8% = 25% per feed. Effect as for background mortality.

Probability per feed vector will acquire Plasmodium infection

Base = 4%

Average lifetime infectious bites per vector lifetime per person as % of value with no intervention, for humans with various protection types

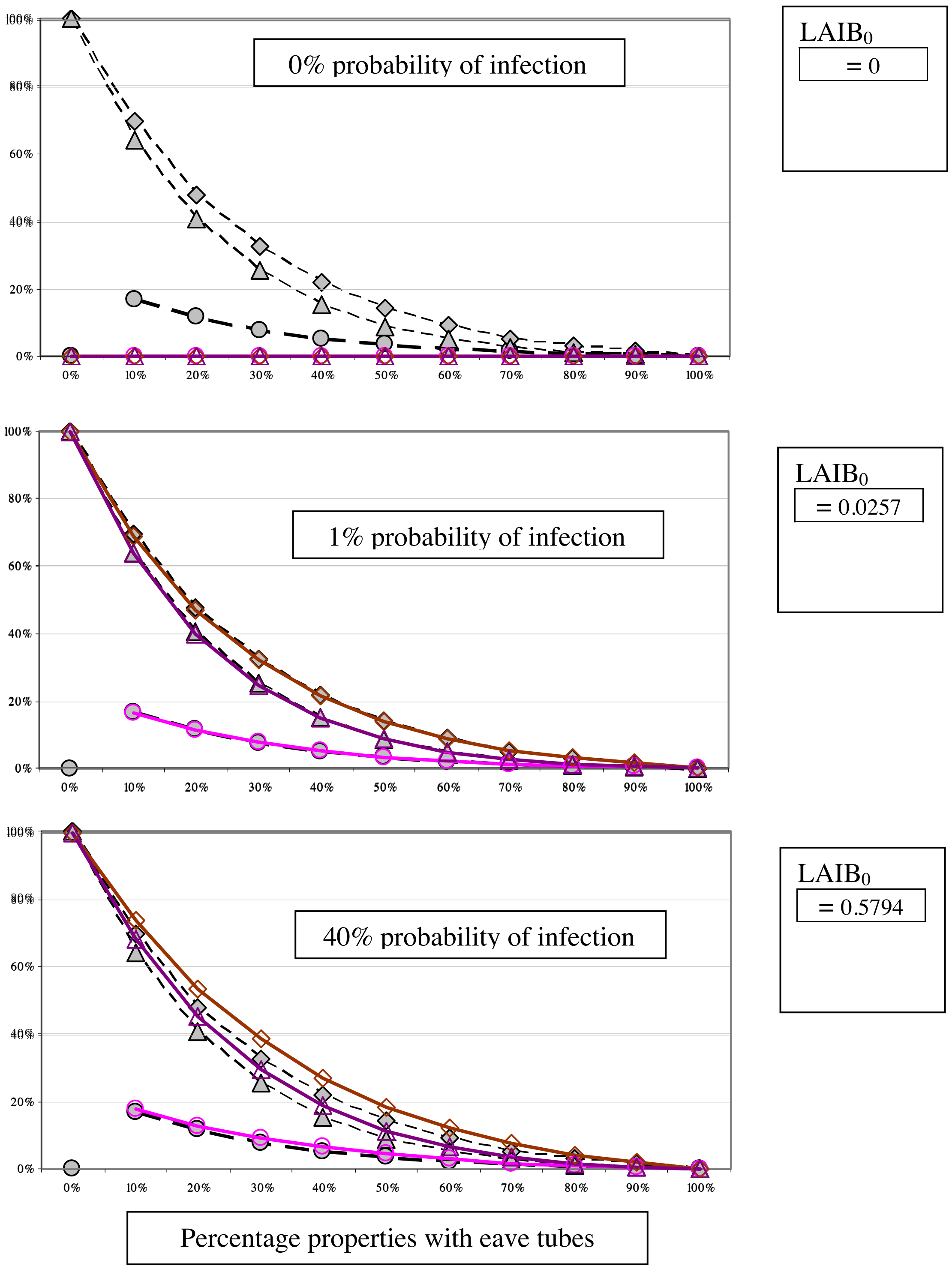

Higher probability of acquiring infection should clearly give higher $LAIB_0$, but should it diminish the proportionate effect of intervention, giving higher VLAIB as well?

Let P = prob of infection (to feeding vector)
Let S = prob survives one cycle
Let M = mortality from intervention
Let μ = background mortality rate
Let τ = time from infection to first infectious bite

Number of infectious bites per lifetime = $\dfrac{PS(1-M)e^{-\mu\tau}}{\left(1-S(1-M)\right)\left(1-S(1-M)(1-P)\right)}$

Does higher probability of acquiring infection give higher VLAIB with given mortality from intervention than lower probability of acquiring infection?

$$\frac{\dfrac{(P+\delta)S(1-M)e^{-\mu\tau}}{\left(1-S(1-M)\right)\left(1-S(1-M)\left(1-(P+\delta)\right)\right)}}{\dfrac{(P+\delta)Se^{-\mu\tau}}{(1-S)\left(1-S\left(1-(P+\delta)\right)\right)}} > \frac{\dfrac{PS(1-M)e^{-\mu\tau}}{\left(1-S(1-M)\right)\left(1-S(1-M)(1-P)\right)}}{\dfrac{PSe^{-\mu\tau}}{(1-S)\left(1-S(1-P)\right)}}$$

$$\rightarrow \frac{(P+\delta)S(1-M)e^{-\mu\tau}(1-S)\left(1-S\left(1-(P+\delta)\right)\right)}{\left(1-S(1-M)\right)\left(1-S(1-M)\left(1-(P+\delta)\right)\right)(P+\delta)Se^{-\mu\tau}} > \frac{PS(1-M)e^{-\mu\tau}(1-S)\left(1-S(1-P)\right)}{\left(1-S(1-M)\right)\left(1-S(1-M)(1-P)\right)PSe^{-\mu\tau}}$$

$$\rightarrow \frac{S\left(1-S\left(1-(P+\delta)\right)\right)}{\left(1-S(1-M)\left(1-(P+\delta)\right)\right)} > \frac{S\left(1-S(1-P)\right)}{\left(1-S(1-M)(1-P)\right)}$$

$$\rightarrow \frac{S\left(1-S\left(1-(P+\delta)\right)\right)\left(1-S(1-M)(1-P)\right)}{\left(1-S(1-M)\left(1-(P+\delta)\right)\right)\left(1-S(1-M)(1-P)\right)} > \frac{S\left(1-S(1-P)\right)\left(1-S(1-M)\left(1-(P+\delta)\right)\right)}{\left(1-S(1-M)(1-P)\right)\left(1-S(1-M)\left(1-(P+\delta)\right)\right)}$$

$$\rightarrow S\left(1-S\left(1-(P+\delta)\right)\right)\left(1-S(1-M)(1-P)\right) > S\left(1-S(1-P)\right)\left(1-S(1-M)\left(1-(P+\delta)\right)\right)$$

$$\rightarrow (1-S+SP+S\delta)\left(1-S(1-M)+S(1-M)P\right) > (1-S+SP)\left(1-S(1-M)+S(1-M)P+S(1-M)\delta\right)$$

$$\rightarrow \begin{pmatrix} (1-S+SP)\left(1-S(1-M)+S(1-M)P\right) \\ +S\delta\left(1-S(1-M)+S(1-M)P\right) \end{pmatrix} > \begin{pmatrix} (1-S+SP)\left(1-S(1-M)+S(1-M)P\right) \\ +S\delta(1-M)(1-S+SP) \end{pmatrix}$$

$$\rightarrow 1-S+MS+SP-MSP > 1-S+SP-M+MS-MSP$$

$\rightarrow SP(1-M) > SP(1-M)-M$ =TRUE as required

EIP, cycles between infection and infectiousness

Base = 3 cycles

Average lifetime infectious bites per vector lifetime per person as % of value with no intervention, for humans with various protection types

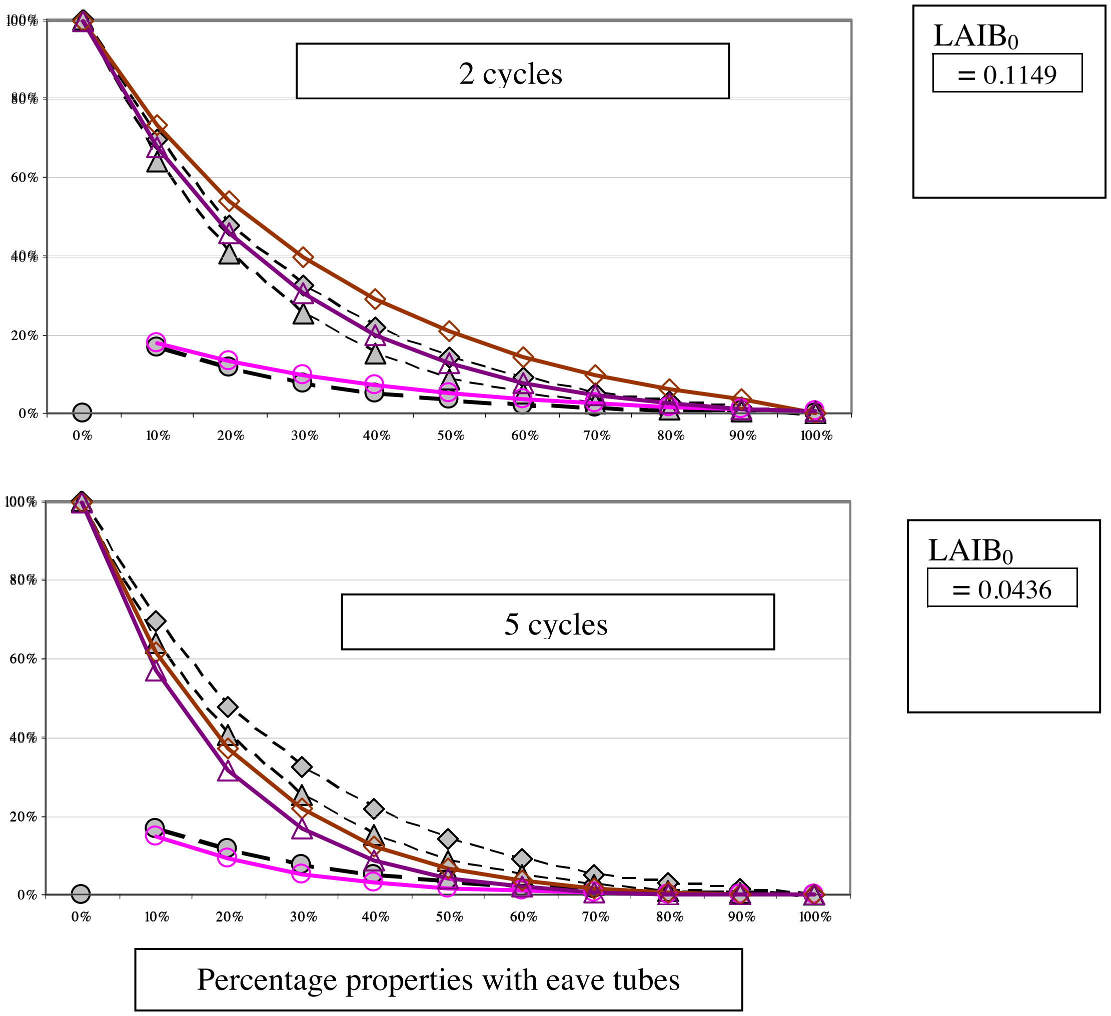

Longer EIP gives lower $LAIB_0$, and should enhance impact of additional per cycle mortality, giving lower VLAIB for higher EIP, as shown above.

LLINS mortality & deflection

Base = 60% deflection from LLIN protected host, 40% mortality pre and post bite if not deflected, 70% coverage in protected properties

Average infectious bites per vector lifetime as % no-treatment value for varying LLIN coverage and deflection (no ET)

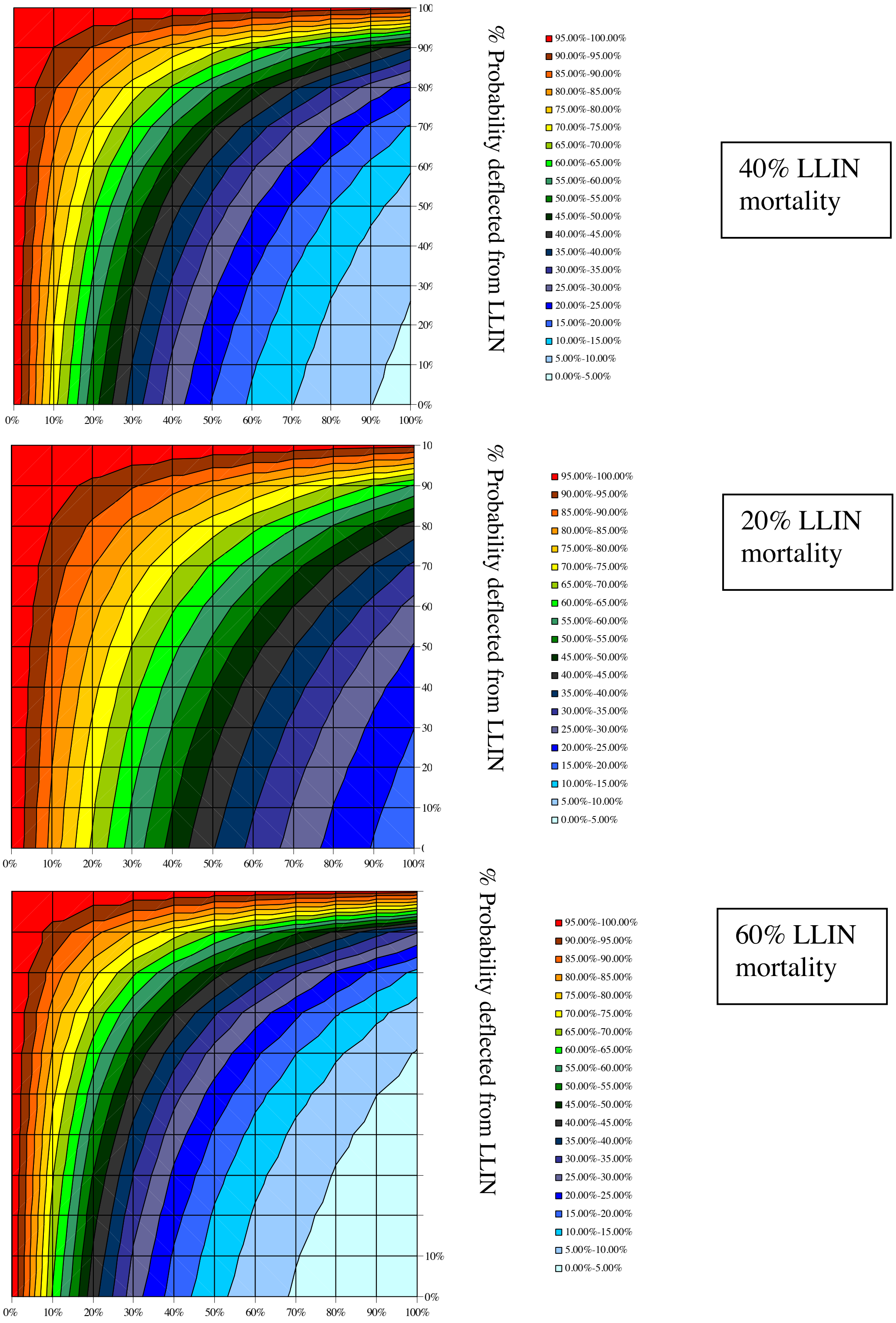

Percentage properties with LLINs

Predictably, LLINs have greater beneficial impact when they generate higher mortality. The negative impact of deflection from LLIN protected people (in the absence of other interventions) is greater for higher LLIN mortality.

Average infectious bites per vector lifetime as % no-treatment value for varying LLIN coverage and deflection (30% ET)

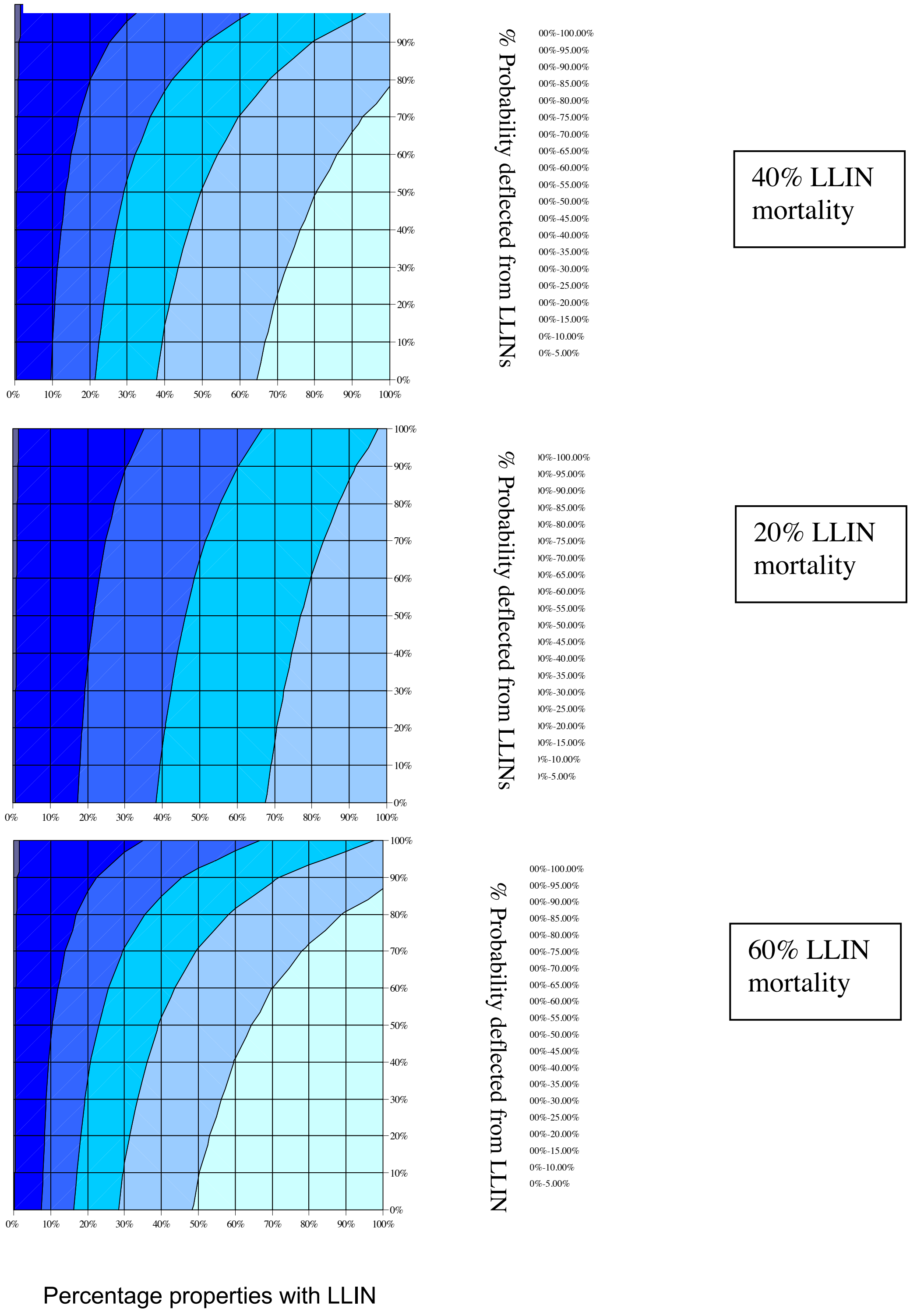

Although a proportion (50%) of vectors deflected away from a bed net protected person are assumed to exit the property, and will then potentially locate and be killed by an ET property, deflection increases the probability that vectors will locate and feed on individuals within a property with LLINS who are not under a LLIN, and therefore increased deflection always reduces the impact of LLINs on VLAIB.

IRS mortality & deflection

Base = 40% mortality, 50% deflection from IRS property

Average infectious bites per vector lifetime as % no-treatment value for varying IRS coverage and deflection (no ET)

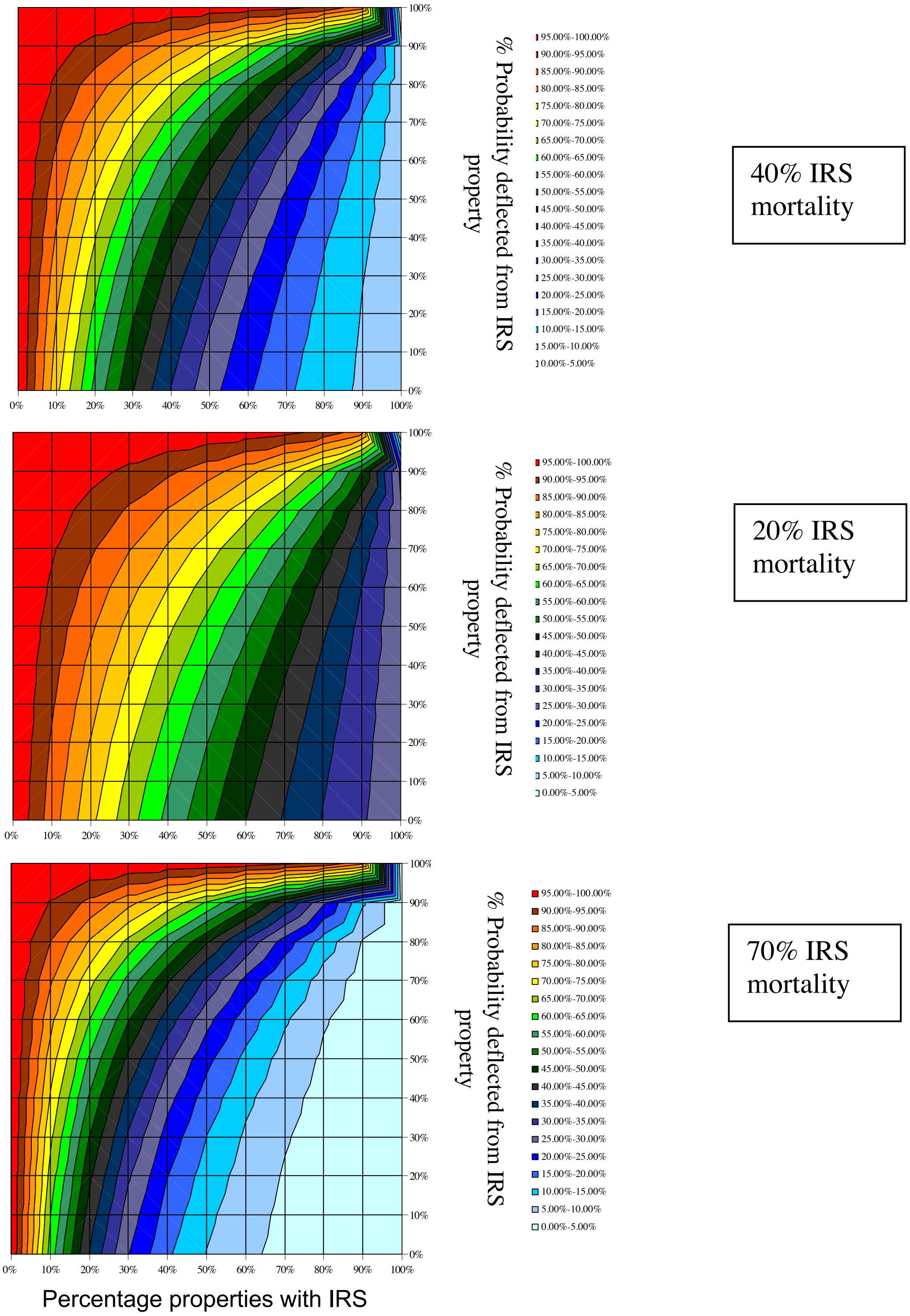

Predictably, IRS has greater beneficial impact when it generates higher mortality. The negative impact of deflection from IRS properties (in the absence of other interventions) is greater for higher IRS mortality.

Average infectious bites per vector lifetime as % no-treatment value for varying IRS coverage and deflection (30% ET)

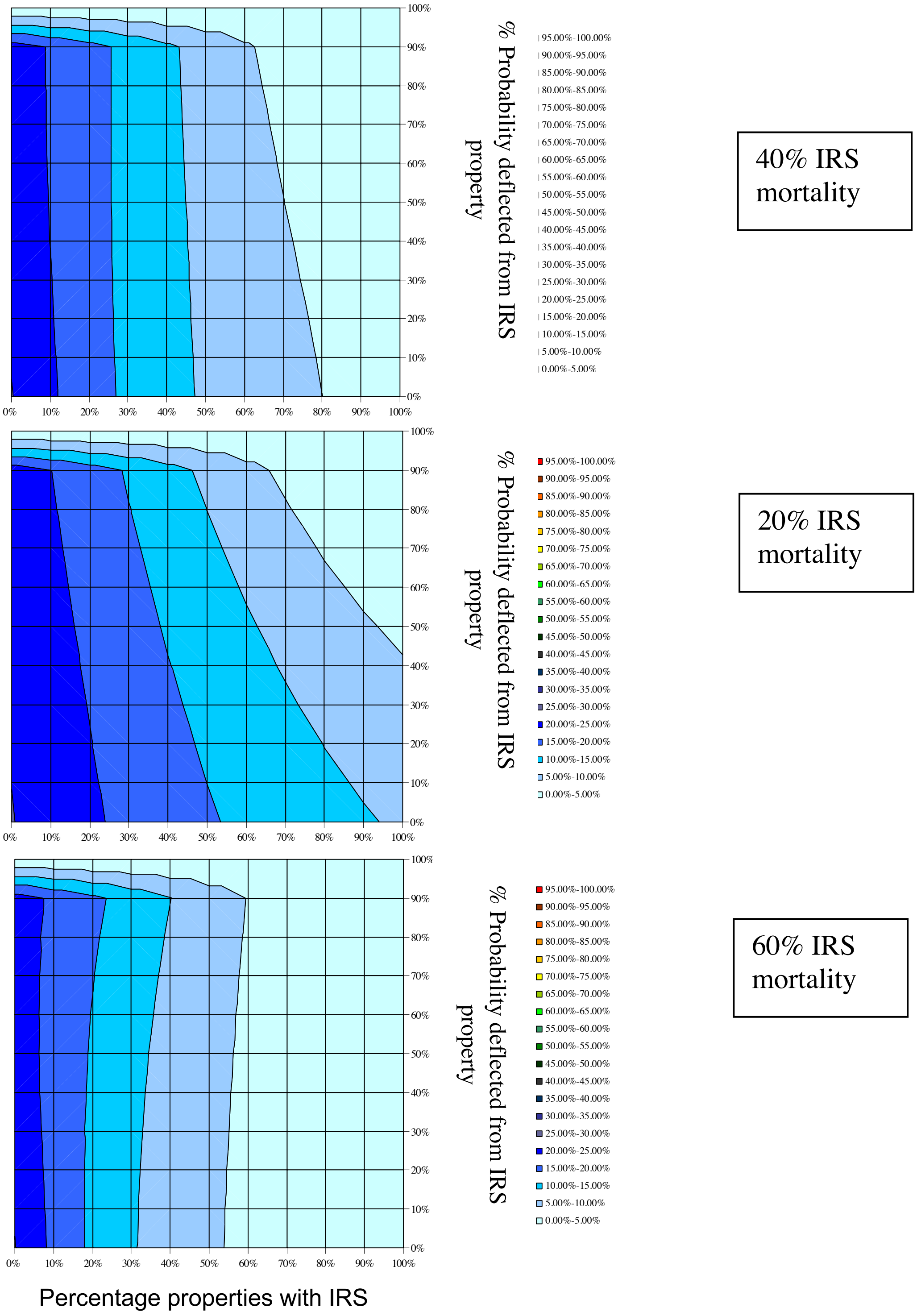

With 30% eave tube coverage, deflection from IRS protected properties can be beneficial if probability of mortality in an IRS treated property is lower than the average probability of mortality across all properties, so assumed IRS mortality value can change the significance of the IRS deflection value, with high IRS mortality making deflection unhelpful, and low IRS mortality making deflection beneficial to reducing VLAIB.
